# Alveolar Macrophages: Development, Respiratory Homeostasis, Signal Pathways, Diseases, and Therapeutic Opportunities

**DOI:** 10.1002/mco2.71031

**Published:** 2026-10-04

**Authors:** Sihan Yang, Ruomeng Li, Chuanjiang Yu, Jing Li, Xikun Zhou

**Affiliations:** ^1^ Department of Biotherapy and Division of Infectious Diseases Cancer Center and State Key Laboratory of Biotherapy West China Hospital Sichuan University Chengdu China; ^2^ State Key Laboratory of Oral Diseases National Clinical Research Center for Oral Diseases Chinese Academy of Medical Sciences Research Unit of Oral Carcinogenesis and Management West China Hospital of Stomatology Sichuan University Chengdu China; ^3^ Institute For Cancer Genetics Columbia University New York USA; ^4^ Laboratory of Pathogen Research West China Hospital Sichuan University Chengdu China; ^5^ Frontiers Medical Center Tianfu Jincheng Laboratory Chengdu China

**Keywords:** alveolar macrophages, alveolar microenvironment, immune response, respiratory diseases

## Abstract

Alveolar macrophages (AMs), as primary immune sentinels in the respiratory tract, are uniquely adapted to tissue‐specific demands shaped by local factors and the physiological environment, maintaining airway homeostasis through the clearance of surfactants and particulates. While AMs exhibit remarkable plasticity shaped by dynamic microenvironmental cues, the traditional M1/M2 paradigm fails to adequately capture this complex heterogeneity, and the specific role of AMs in the pathogenesis of pulmonary diseases, as well as AM‐centric therapies, remain to be fully elucidated. This review systematically synthesizes the current understanding of AM ontogeny, homeostatic regulation, and their signaling pathways in lung homeostasis and pathologies. Furthermore, we propose a refined conceptual framework categorizing AMs into five distinct functional subtypes to better capture their in vivo plasticity. Then, pathological shifts in the lung microenvironment that drive the pathogenesis of diverse pulmonary disorders, including asthma, chronic obstructive pulmonary disease, acute lung injury, and pulmonary fibrosis, and trigger dysregulated transcriptional and metabolic reprogramming in AMs, are comprehensively discussed. Finally, we explore emerging therapeutic avenues, including metabolic modulation, trained immunity, and autophagy–apoptosis balance restoration. By addressing challenges in precisely regulating AM function, this overview provides a concise yet comprehensive framework for advancing AM‐centric therapies in respiratory medicine.

## Graphical abstract

Alveolar macrophages: development, respiratory homeostasis, signal pathways, diseases, and therapeutic opportunities: During embryonic development, fetal progenitors migrate to the developing lungs via a CX3CR1‐dependent mechanism to establish the TrAM pool. Conversely, under conditions of aging, infection, or structural injury (e.g., fibrosis), bone marrow‐derived circulating monocytes are recruited to the alveolar niche and differentiate into MoAMs. Within the alveolar microenvironment, the maturation and functional maintenance of AMs are strictly governed by key niche‐derived cytokines, primarily GM‐CSF, M‐CSF, and TGF‐β, secreted by surrounding structural cells (e.g., Type 1/2 pneumocytes and fibroblasts). As central regulators of respiratory homeostasis, AMs orchestrate the delicate balance between immune defense and tissue repair. However, pulmonary diseases often trigger a critical shift from homeostatic TrAMs to pathological MoAMs. Consequently, modulating AM phenotypes and metabolic states presents a promising clinical avenue. Emerging AM‐centric strategies, including immunotherapy, repolarization, targeted transplantation, metabolic interventions, and dietary approaches, aim to restore the TrAM/MoAM balance and facilitate lung regeneration.

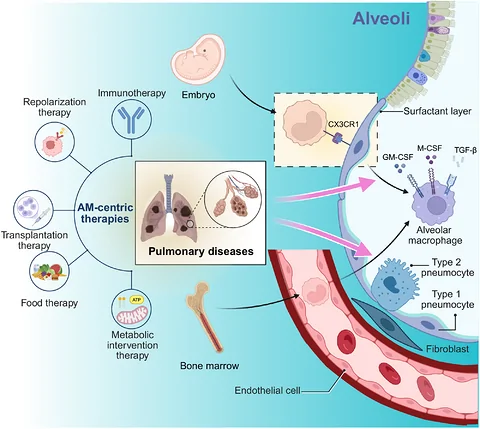

## 1 Introduction

The respiratory tract is continuously exposed to a vast array of airborne pathogens, allergens, and environmental particulates. Situated at the air–tissue interface, alveolar macrophages (AMs) act as the lung's primary innate immune sentinels and first line of defense [1, 2, 3]. As specialized residents of this niche, these cells possess unique adaptations tailored to the specific demands of the local alveolar microenvironment. In a healthy lung, AMs are essential for maintaining immune tolerance and airway homeostasis, a function largely achieved through the continuous clearance of pulmonary surfactant, apoptotic cells, and inhaled debris without triggering unnecessary inflammation [4, 5, 6].

Historically, AMs were conceptualized as a homogeneous cellular population. However, recent ontogenetic and transcriptomic advances have fundamentally revised this paradigm, unveiling substantial heterogeneity in their origins and functions. It is now established that the AM pool comprises two distinct groups: tissue‐resident AMs (TrAMs) that originate from embryonic monocytes and are maintained through local self‐renewal, and monocyte‐derived AMs (MoAMs) that are recruited from the bloodstream [7, 8]. During respiratory infections or tissue injury, MoAMs undergo rapid expansion, contributing significantly to inflammation and disease progression [9]. Beyond their diverse origins, it has become evident that the local microenvironment, through specific signaling molecules and metabolic cues, profoundly controls AM functional plasticity. This biological complexity and phenotypic flexibility transcend the traditional M1/M2 macrophage dichotomy [10]. For instance, during sepsis, alveolar epithelial Type II (AT2)‐derived exosomal lncRNA Rmrp degrades AM *Pfkfb3*, forcing these cells into a unique state of metabolic exhaustion and immune paralysis. This adaptation is hard to be categorized into the rigid M1/M2 phenotype [11].

The disruption of this delicate microenvironmental equilibrium precipitates severe pathological consequences [12]. Under disease conditions, AMs undergo profound metabolic shifts and dysregulated phenotypic transitions. For example, impaired AM lipid catabolism triggers pulmonary alveolar proteinosis (PAP), aberrant metabolic reprogramming impairs phagocytosis in chronic obstructive pulmonary disease (COPD), and excessive transforming growth factor‐beta (TGF‐β) secretion orchestrates aberrant tissue remodeling in pulmonary fibrosis [13, 14, 15]. Treatments for these diseases largely overlook AM dysfunction and often yield suboptimal clinical outcomes or severe immunosuppressive effects, highlighting a critical gap in the therapeutic arsenal. Specifically, broad‐spectrum corticosteroids often provoke systemic immunosuppression, while standard antifibrotics like pirfenidone target downstream fibroblasts, entirely neglecting upstream macrophage‐driven signals [16, 17]. Therefore, a deeper understanding of the precise mechanisms underlying AM dysfunction is urgently required to develop targeted therapies that maximize efficacy while minimizing off‐target adverse effects [18].

This review first traces the ontogeny and heterogeneity of AMs, distinguishing tissue‐resident, monocyte‐derived, homeostatic, disease‐associated, and memory‐like states. It then examines how AMs maintain alveolar homeostasis through self‐renewal, host defense, inflammation resolution, surfactant metabolism, efferocytosis, tissue repair, metabolic adaptation, and crosstalk with neighboring cells. Next, we organize the principal homeostatic and stress‐responsive signaling pathways that govern AM identity and function, before evaluating their context‐dependent roles across major respiratory diseases. Finally, we compare emerging AM‐targeted therapeutic strategies and clinical trials and discuss the key challenges for translating AM biology into precise, disease‐ and state‐specific interventions.

## 2 The Development of AMs

### 2.1 The Ontogeny of AMs

The developmental origins of AMs have been a focal point of recent research, revealing a complex interplay between embryonic and hematopoietic progenitors. Over five decades ago, van Furth proposed that all tissue‐resident macrophages (TRMs) arise from and are replenished by circulating monocytes [19]. However, this hypothesis was swiftly refuted by studies demonstrating that many TRMs, including AMs, establish themselves during embryonic development and persist without monocyte input [20, 21]. Lung transplantation studies further clarified that human AMs originate from two distinct sources: embryonic precursor cells established during fetal development (TrAMs) and circulating monocytes recruited postnatally (MoAMs) [22, 23, 24, 25, 26]. This dual origin underscores AMs’ adaptability to both homeostatic and pathologic conditions.

Mouse and human AMs are seeded by fetal progenitors during perinatal lung development and, under noninflammatory conditions, persist largely through local self‐renewal with minimal input from circulating monocytes [6, 27, 28]. During aging [28, 29], infection [30, 31, 32], fibrosis [8, 33, 34], and lung regeneration [35], the proportion of circulating monocytes that develop from embryonic stem and progenitor cells in the bone marrow as precursors of AMs increases [27], differentiating into MoAMs to meet the increased demand for immune responses [27]. This ontogenetic shift from TrAM dominance in homeostasis to MoAM expansion in pathology highlights the balance between tissue maintenance and immune defense, with each subset exhibiting distinct functional phenotypes: TrAMs prioritize surfactant clearance and anti‐inflammatory homeostasis, while MoAMs mediate proinflammatory responses to insults [6, 36, 37].

The transition from blood monocytes to functional AMs involves distinct molecular reprogramming. CD14^+^CD16^−^ blood monocytes, originating from hematopoietic stem and progenitor cells (HSPCs), migrate into the lung interstitium and differentiate into interstitial macrophages (IMs) and AMs. During maturation, lung monocytes sequentially upregulate the expression of CD206 and CD169 [38]. Compared with TrAMs, mature MoAMs have a genetic proinflammatory signature induced by T lymphocytes [39] and IFN‐induced macrophages [38], aligning with their role in acute immune responses [40, 41, 42]. In this case, HLA‐DRhi monocytes originating from CD14^+^CD16^−^ monocytes actively migrate into lung tissue, where they serve as transitional cells for the differentiation of human lung macrophages derived from blood monocytes [38].

Furthermore, CD34^−^Lin^−^CD116^+^CD64^−^CD115^+^ macrophage progenitors originate from the fetal liver and can enter the lung through a process dependent on the chemokine receptor CX3CR1 [27, 43]. Upon exposure to tissue‐derived macrophage colony‐stimulating factor (M‐CSF) and granulocyte–macrophage colony‐stimulating factor (GM‐CSF), these cells differentiate into mature human AMs [27]. CX3CR1 is expressed on fetal AMs precursors, and its ligand, CX3CL1, is also expressed in human fetuses and lungs [44, 45]. Upon entering the lung, CD116^+^CD64^−^ macrophage precursors increase the expression of CD64 and other surface markers, such as CD206 and CD169, a process that aligns with their capacity to develop into mature macrophages and remove surfactant from the alveoli. At this point, fetal liver AMs precursor cells express receptors of critical macrophage cytokines, including GM‐CSF [27], M‐CSF [27], and TGF‐β [46]. In establishing the alveolar ecological niche, fetal CD116^+^CD64^−^ precursor cells of human AMs express higher levels of proliferation‐related genes than CD14^+^ blood monocytes, enabling them to occupy the niche more rapidly. After maturation, AMs derived from CD116^+^CD64^−^ cells behave distinctly; CD116^+^CD64^+^ macrophages, despite having an AM surface phenotype, cannot effectively degrade and metabolize lung surface‐active substances. This functional limitation suggests that fetal liver progenitors follow a stepwise differentiation pathway: CD116^+^CD64^−^ → CD116^+^CD64^+^ fetal monocytes → mature AMs, with each stage governed by distinct cytokine signaling [27]. This ordered development ensures the establishment of functional TrAMs to maintain alveolar balance.

The functional implications of these ontogenetic pathways are profound. TrAMs are critical for baseline lung homeostasis, clearing inhaled particles and dead cells while minimizing inflammation. During severe lung injury, the affected cells are often replenished by macrophages derived from circulating blood monocytes (Figure 1). Human embryonic progenitor cells have the potential to mitigate organ damage and facilitate tissue repair in injured lungs, making them promising candidates for regenerating protective macrophages. Elucidating the embryonic and cellular origins and developmental pathways of human AMs provides new insights into their impact on human AM specificity and function, facilitating the development of AM‐based therapeutics for human lung diseases.

**FIGURE 1:**
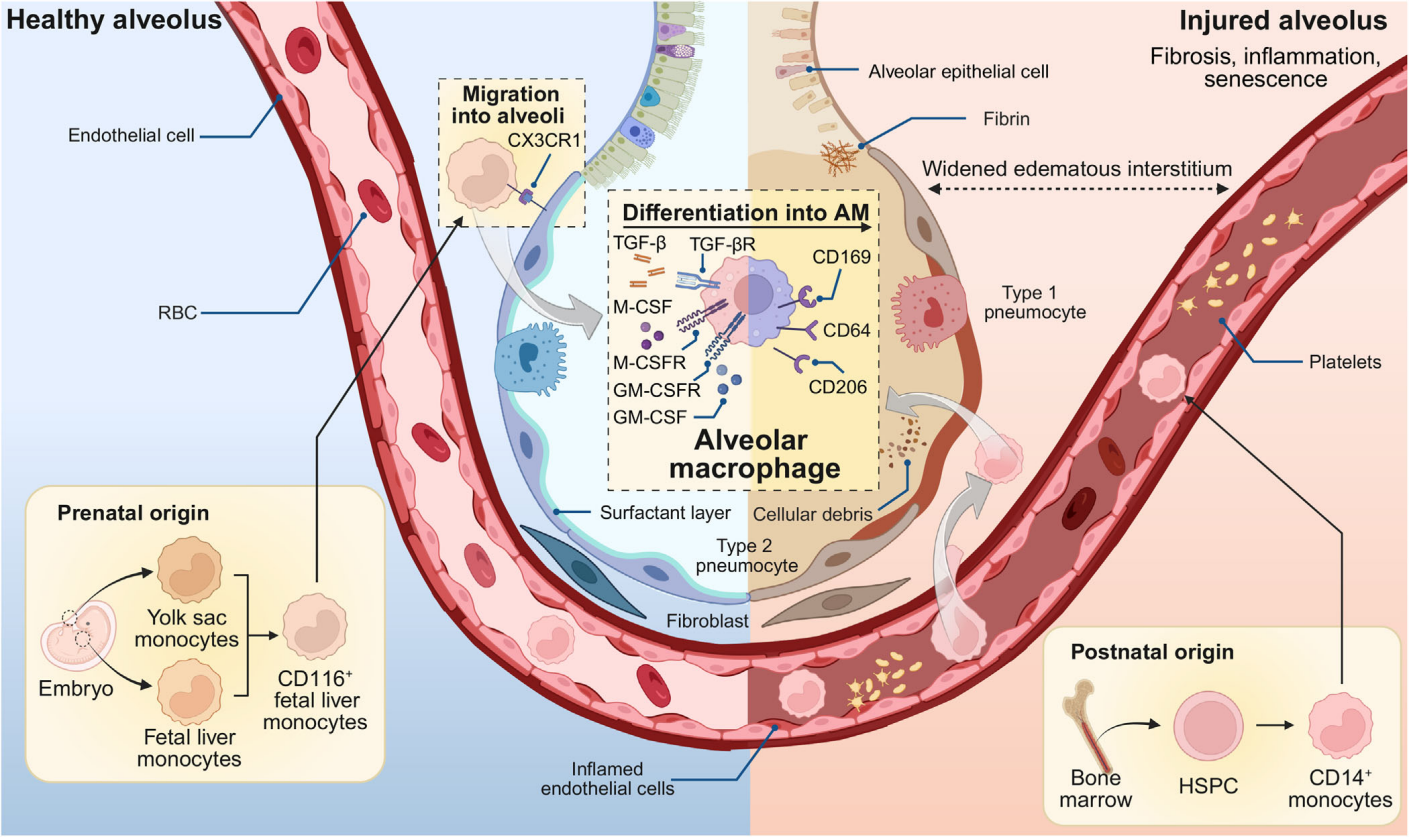
Schematic illustration of the origin of AMs. During embryonic development, circulating CD116^+^ fetal progenitor cells originating from the yolk sac or fetal liver migrate to the lungs in a CX3CR1‐dependent manner to differentiate into TrAMs prenatally and occupy alveolar ecological niches during the perinatal period of lung growth. Without inflammation, TrAMs self‐renew locally (no input from circulating monocytes). During aging, infection, fibrosis, and lung regeneration, the proportion of circulating CD14^+^ monocytes (derived from bone marrow HSPCs) that serve as MoAM precursors increases in injured alveoli. Key cytokines (GM‐CSF, M‐CSF, TGF‐β) promote AM differentiation, with precursors upregulating CD64, CD206, and CD169 to become functional AMs. The balance between TrAMs (homeostasis) and MoAMs (pathology) is highlighted.

### 2.2 The Heterogeneity of AMs

Historically, AM polarization has been conceptualized through a classically activated M1 phenotype, characterized by robust proinflammatory cytokine secretion and a metabolic shift toward aerobic glycolysis, and a M2 paradigm, which relies on oxidative phosphorylation (OXPHOS) to drive anti‐inflammatory and tissue‐repairing functions [47]. Although this dichotomy provides a useful in vitro heuristic, emerging evidence demonstrates that in vivo AM states are far more heterogeneous and context dependent than a binary classification can capture, rendering the strict M1/M2 paradigm obsolete [48].

For instance, under steady states, AM homeostasis depends primarily on OXPHOS and lipid metabolism regulated by the natural killer receptor protein 1B (NKR‐P1B) signal, rather than glycolysis [49]. Furthermore, during sepsis, instead of simply transitioning to an M2 phenotype, AMs enter a unique state of metabolic exhaustion and immune paralysis, which fits neither M1 nor M2 profiles. This distinct state is driven by AT2‐derived exosomal lncRNA Rmrp, which mediates *Pfkfb3* degradation, severely impairing AM glycolysis and inducing profound immune tolerance [11].

Therefore, inspired by rapid updates to single‐cell and spatial omics datasets, we propose a multidimensional framework that delineates AM heterogeneity across three specific contexts to replace these outdated models: ontogenic origins (TrAMs vs. MoAMs), steady‐state homeostasis (proliferating AMs and senescent AMs), and disease‐associated reprogramming (inflammatory AMs, repair/regulatory AMs, and memory AMs).

#### 2.2.1 TrAMs Versus MoAMs

TrAMs and MoAMs are fundamentally distinguished by their ontogeny and subsequent functional adaptations. Under homeostatic conditions, TrAMs are predominantly established from fetal liver progenitors during the perinatal period [27]. Once established within the alveolar niche, they maintain baseline homeostasis, prioritizing surfactant clearance and anti‐inflammatory functions, through intrinsic local self‐renewal, with negligible input from mature circulating monocytes [6, 27, 28, 36, 37]. However, while TrAMs exhibit such active transcriptional subversion during allergic inflammation, their autonomous maintenance capacity remains highly vulnerable to severe pathogenic insults. During bacterial pneumonia, surviving TrAMs undergo infection‐induced cellular senescence, upregulating cell‐cycle arrest genes (e.g., Cdkn2a) and exhibiting diminished proliferative capacity [50]. Similarly, severe viral pneumonia triggers catastrophic apoptosis, leading to a profound collapse of the resident network [51].

In stark contrast to the homeostatic dominance of embryonic TrAMs, conditions such as aging [28, 29], fibrosis [8, 33, 34], and severe infections [30, 31, 32], trigger a profound ontogenetic shift favoring the expansion of MoAMs [27]. Originating from adult HSPCs, these recruited monocytes exhibit a unique genetic and metabolic phenotype reflecting their distinct developmental lineage. Compared with TrAMs, MoAMs inherently possess a proinflammatory signature aligned with their role in acute immune responses [40, 41, 42]. Furthermore, they are phenotypically distinguished by the sustained high expression of specific lipid‐associated genes (CD11b, ApoE, GPNMB, Trem2) and undergo metabolic reprogramming toward enhanced glycolysis [50, 52]. In sterile contexts, their terminal maturation is intricately governed by a shift from Notch to Wnt/β‐catenin signaling, and their long‐term survival relies heavily on localized paracrine circuits, particularly the ApoE‐mediated M‐CSF signaling axis, rather than autonomous proliferation [52, 53].

When the embryonic TrAM pool is irreversibly decimated by severe viral insults, these monocyte‐derived cells exhibit the ultimate capacity to take over the long‐term maintenance of the alveolar niche altogether. Under such conditions, recruited monocytes undergo a highly orchestrated differentiation trajectory. Initially, infiltrating monocytes rapidly differentiate into a unique Slamf9^+^ macrophage subset characterized by the coexpression of Spp1, Siglec1, and Il10. Unlike the highly vulnerable TrAMs, these Slamf9^+^ cells are apoptosis resistant, highly proliferative (Mki67^+^), and serve as primary drivers of viral clearance by actively recruiting neutrophils. Upon viral clearance, the surviving Slamf9^+^ subset transitions into Trem2^+^ macrophages (Trem2^hi^Spp1^hi^) specialized in mitigating postinflammatory fibrosis. As tissue repair progresses, these cells further evolve into Fbp1^+^ macrophages (Fbp1^hi^), featuring a proliferative subpopulation (Fbp1^+^Mki67^+^) that spatially colocalizes with the residual alveolar niche to aggressively replenish the depleted macrophage pool [51]. Collectively, this dynamic interplay highlights a critical ontogenetic division of labor: while TrAMs are hardwired for baseline homeostasis, MoAMs leverage their distinct developmental plasticity to fundamentally reconstitute and upgrade the alveolar network when endogenous defense fails.

#### 2.2.2 Homeostatic Subsets

Traditionally, homeostatic TrAMs were considered a homogeneous population. However, recent advances in single‐cell RNA sequencing (scRNA‐seq) and spatial transcriptomics reveal significant transcriptional heterogeneity within the steady‐state alveolar niche, demonstrating that TrAMs compartmentalize into distinct functional states to independently sustain their population [1, 2]. This local turnover relies on balancing cellular renewal and attrition, driven by two distinct subsets: the actively dividing proliferating AMs for replenishment and the terminally exhausted senescent AMs destined for clearance.

Proliferating AMs maintain TrAM pool stability (>90% under homeostasis) through Wnt/β‐catenin‐regulated self‐renewal driven by GM‐CSF niche signals, and this actively proliferating state is transcriptionally validated by cell cycle progression and the expression of canonical proliferation markers Top2a and Mki67 [54]. The specific mechanisms governing this process are comprehensively detailed in the upcoming section on “Signaling pathways.”

Senescent AMs arise primarily from embryonically‐derived TrAMs that progressively lose proliferative capacity with aging or following severe/recurrent infections (e.g., sublethal *Streptococcus pneumoniae* [*S. pneumoniae*]), displaying elevated SA‐β‐Gal activity, reduced self‐renewal, and metabolic decline [50, 55]. Phenotypically, they upregulate major histocompatibility complex (MHC) II (driven by IFN‐γ from T cells), yet exhibit impaired pathogen clearance and respiratory homeostasis, contributing to pneumonia susceptibility in the elderly and chronic inflammation in COPD [56, 57]. Transcriptionally, they adopt a distinctive proinflammatory profile with elevated CCL2, IFN‐β, interleukin (IL)‐10, IL‐12p40, tumor necrosis factor (TNF)‐α, and macrophage migration inhibitory factor (MIF), fostering a chronic inflammatory alveolar microenvironment [58]. Senescent AMs are gradually outcompeted (within 2–3 weeks postinfection) by MoAMs to repopulate the alveolar niche [50].

#### 2.2.3 Disease‐Associated Subsets

During pulmonary pathogenesis, external microenvironmental cues, such as pathogen‐associated molecular patterns (PAMPs), danger signals, and localized cytokines, disrupt alveolar homeostasis, inducing AMs to reprogram into highly specialized, disease‐associated subsets [59]. Based on the sequential phases of the disease pathology, these subsets are categorized into inflammatory AMs that initiate acute host defense, repair/regulatory AMs that orchestrate tissue resolution or drive pathological fibrosis, and memory AMs that mediate trained immunity [5, 60].

Although characterized by an M1‐like elevated proinflammatory transcriptional profile (including TNF‐α, IL‐12p40, C–C motif chemokine ligand 2 [CCL2], IFN‐β, and MIF), inflammatory AMs exhibit a distinct, context‐dependent metabolic and phenotypic reprogramming that fundamentally diverges from the classical M1 macrophage paradigm [61]. For example, in the context of *Mycobacterium tuberculosis* (*Mtb*) infection, inflammatory AMs exhibit a distinct phagocytic phenotype, transcriptionally featuring the robust expression of *Nos2* and the significant upregulation of a specific proinflammatory gene signature including *Clec4e* and *Saa3* [54].

Besides, challenging the conventional paradigm that TrAMs are immunoregulatory and resilient to inflammatory reprogramming, recent evidence highlights their profound plasticity during acute Type 2 immunity. Mechanistically, IL‐33‐activated Group 2 innate lymphocytes (ILC2s) secrete IL‐13, engaging IL‐4Rα on TrAMs to robustly induce IRF4. As a molecular switch, IRF4 suppresses peroxisome proliferator‐activated receptor (PPAR)‐γ, silencing homeostatic identity genes (*SiglecF*, *Marco*, *Krt19*). Concurrently, IRF4 drives an alternatively activated macrophage state (upregulating CD11c/b, CD2, *Arg1*, *Chil3*, *Retnla*) and activates cell‐fusion (*Ocstamp*, *Dcstamp*, *Kcnn4*) and chemokine (*Ccl17*, *Ccl24*) regulons. Ultimately, this cascade promotes multinucleated giant cell formation and leukocyte recruitment, transforming TrAMs into active drivers of Type 2 inflammation [62].

Repair/regulatory AMs suppress excessive alveolar inflammation through efferocytosis and PPAR‐dependent oxidative metabolism, while promoting alveolar epithelial regeneration via the osteopontin–CD44 axis. They are essential for acute respiratory distress syndrome (ARDS) resolution, but their dysregulation contributes to aberrant remodeling in pulmonary fibrosis [48]. For instance, a profibrotic, immature AM subset localized to fibrotic niches, termed Spp1^+^Macs, has been identified in silicosis. These cells increase progressively during the development of silicosis, and the key gene Pla2g7 (encoding Lp‐PLA2) is highly expressed in this subset. Mechanistically, Lp‐PLA2 disrupts cardiolipin metabolism and impairs mitophagy, driving the persistence of the immature profibrotic macrophage phenotype [63].

Moreover, the marked upregulation of TREM2 in MoAMs was observed in both animal models and patients with idiopathic pulmonary fibrosis (IPF), which sustained a profibrotic phenotype in a sphingomyelin‐driven manner. These TREM2^+^ MoAMs were protected from apoptosis, and exacerbated pulmonary fibrosis by secreting profibrotic mediators and impairing alveolar epithelial regeneration [64].

Memory AMs exhibit a dual functional identity, trained or tolerogenic, shaped by context‐dependent priming pathways. Trained memory AMs provide broad, antigen‐independent protection, programmed via either CD8^+^ T cell‐dependent local viral infections or T cell/IFN‐γ‐independent systemic circuits like gut–lung axis post‐Bacillus Calmette‐Guérin (BCG) vaccination [65, 66]. Conversely, tolerogenic memory AMs display long‐term immunoparalysis, impairing postinfection bacterial clearance and increasing susceptibility to hospital‐acquired pneumonia [67]. This dual identity is driven by widespread epigenetic remodeling (DNA hypomethylation, open chromatin, and enriched IRF/NF‐κB/C/EBP/STAT6/ATF3 motifs), which can even be induced by noninfectious local cues (e.g., native mucus) to cause impaired efferocytosis [68]. However, the differentiation dynamics, tissue distribution, and convergence of these pathways remain poorly defined.

This taxonomy accurately reflects spatial coordination and temporal state transitions. Despite its convenience as a label for pro‐ or anti‐inflammatory bias, the M1/M2 dichotomy tells us little about how AMs actually behave in the tissue. Embracing the profound heterogeneity of AMs driven by ontogeny, distinct metabolic reprogramming, and spatial niche cues through high‐resolution, multiomic taxonomies is essential for advancing AM‐targeted therapies in lung diseases.

## 3 Homeostasis Regulation by AMs

The unique environment of the alveoli shapes AMs, and in turn, AMs play a crucial role in maintaining alveolar homeostasis through a balanced network of cytokine signaling, self‐renewal, and cell–cell interactions. This interplay ensures AMs adapt dynamically to both physiological and pathological conditions while preserving lung integrity.

### 3.1 Self‐Renewal/Maintenance of AMs

AMs originate from fetal liver monocytes that seed the alveolar tissue and differentiate into macrophages. When these AMs are depleted, the cellular niche is replenished by the in situ proliferation of the remaining pool rather than recruitment and differentiation of circulating monocytes [9]. This self‐renewal capacity is critical for sustaining AM numbers under homeostatic conditions. Mechanistically, AM self‐renewal involves stem cell‐like proliferation and the maintenance of a unique transcriptional identity that distinguishes them from other macrophage subsets [69]. The Wnt/β‐catenin pathway, combined with GM‐CSF, TGF‐β, and PPAR‐γ signaling, synergistically sustains AM self‐renewal under homeostasis.

Liver kinase b1 (Lkb1) is a serine‐threonine kinase and tumor suppressor that is involved in several processes and is critical for macrophage function [70, 71]. The deletion of Lkb1 from CD11c^+^ cells significantly reduced the abundance of AMs in the lungs. Mice lacking Lkb1 displayed exacerbated disease susceptibility during infection with *Staphylococcus aureus* (*S. aureus*) and allergic inflammation. These mice exhibited excessive accumulation of neutrophils and more severe lung pathology during airway *S. aureus* infection. Lkb1 serves as a critical regulator, ensuring the self‐renewal and immune function of AMs [72].

In addition to the key regulators mentioned above that influence the proliferation and immune function of AMs, metabolites such as 12‐hydroxyeicosatetraenoic acid (12‐HETE), prostaglandin E2 (PGE2), and core‐binding factor beta (CBFβ) equally affect the maintenance and self‐renewal of AMs. In Alox15−/− mice, the production of 12‐HETE by neonatal neutrophils is critical for the self‐renewal and maintenance of AMs during lung development. The absence of 12‐HETE leads to a substantial decrease in AMs in the lungs of adult mice. Disruption of the AM population increases the susceptibility of mice to lipopolysaccharide (LPS)‐induced acute lung injury (ALI). Additionally, this condition makes mice more prone to lung infections caused by *influenza A virus* or *SARS‐CoV‐2* [55]. Exogenous PGE2 suppresses AM proliferation via the E prostanoid receptor 2 (EP2)–cAMP signaling pathway. In addition, EP2 knockout mice exhibit increased numbers of AMs, which are resistant to the inhibition of proliferation induced by PGE2 and bronchoalveolar lavage fluid (BALF) from aged mice [73]. A decrease in CBFβ was observed in the cytosol of AMs from aged mice. A similar senescence‐like phenotype was found in human AMs. AMs from aged mice showed impaired self‐renewal ability in response to GM‐CSF [74]. Moreover, AM differentiation, self‐renewal, and homeostatic functions require intact oxygen‐sensing capacity [75].

### 3.2 Immune Defense Functions of AMs

In the alveoli and larger airways, AMs are the sentinel cells crucial for maintaining homeostasis, resolving inflammation, clearing bacteria, and pathogens, and thereby preventing invasive infection [76].

#### 3.2.1 Phagocytosis and Clearance of Pathogens/Particles

AMs are gatekeepers in airways that execute respiratory antimicrobial defense through their robust phagocytic and microbicidal capacities [59, 77]. Positioned at the delicate air–tissue interface, they function as highly dynamic sentinels rather than passive residents. They crawl between alveoli to engulf and destroy pathogenic bacteria, a process that simultaneously dampens the activation of secondary immune cascades, thereby preventing pathological inflammation [78].

Mechanistically, lymphocyte function‐associated antigen 1‐driven crawling enables AMs to continuously scan the alveolar surface. Upon microbial contact, targets are rapidly recognized via lung opsonins (e.g., surfactant protein [SP]‐A, SP‐D) and scavenger receptors (e.g., macrophage receptor with collagenous structure [MARCO]), then internalized and destroyed within mature phagolysosomes by hydrolytic enzymes and reactive oxygen species (ROS) [5, 78, 79, 80, 81]. This cloaking mechanism allows AMs to clear ubiquitous bacteria without provoking lung inflammation.

However, during respiratory infection by *Mtb*, AMs were demonstrated to adopt a pathogen‐permissive phenotype, whereas IMs remained pathogen restrictive [61]. This underscores that the very mechanism designed for silent clearance can be exploited by cunning intracellular pathogens to evade destruction. In turn, the microbiota can also deploy a variety of mechanisms via delivering circRNAs or interacting with intracellular proteins like etoposide‐induced protein 2.4 homolog (EI24) and Nod‐like receptor family CARD domain‐containing 5 (NLRC5) to modulate host immune responses, thereby further shaping the functional outcomes of pathogen clearance [82, 83, 84, 85].

Confronted with other resilient fungal targets, such as *Aspergillus fumigatus* spores, AMs deploy distinct recognition and clearance pathways. Fungal phagocytosis is initiated when Dectin‐1 recognizes exposed β‐glucans, triggering actin‐dependent engulfment. As thick‐walled spores demand prolonged intracellular processing and risk runaway inflammation, AMs undergo EGR2‐mediated epigenomic rewiring that sustains Dectin‐1 expression and phagocytic capacity [86]. Furthermore, AMs achieve complete fungal clearance via adiponectin‐driven LC3‐associated phagocytosis. Crucially, this pathway combines rapid degradation with suppressed proinflammatory signaling, preserving lung tolerance [87].

However, this finely tuned system is highly susceptible to viral subversion. Viral interference manifests in two distinct ways. On one hand, respiratory viruses like influenza A (IAV) induce a profound “immune paralysis.” IAV‐driven IFN‐γ signaling halts AM motility, stripping them of their patrolling capabilities and causing a catastrophic failure in secondary bacterial clearance. Fortunately, gut microbiota‐derived signals can epigenetically rescue this vulnerability by reprogramming AM chromatin to re‐express genes essential for motility and immune patrol [5, 88, 89]. On the other hand, certain systemic viruses actively hijack the phagocytic apparatus. African swine fever virus (ASFV) hyper‐activates CD14‐dependent phagocytosis in AMs. This excessive engulfment of infected apoptotic bodies floods the cytosol with viral DNA, continuously triggering cyclic GMP–AMP synthase (cGAS)/stimulator of interferon genes (STING) signaling to fuel a lethal cytokine storm and accelerate viral dissemination [90]. Indigestible environmental particulates (e.g., diesel exhaust particles and silica) further expose a fundamental mechanical limitation in AM‐mediated clearance. PM2.5 uptake via the Msr1 receptor drives a severe acute proinflammatory response [91].

#### 3.2.2 Induction and Resolution of Inflammation

Resolution biology is characterized by (i) production of proresolving mediators and anti‐inflammatory cytokines and (ii) attenuation of leukocyte infiltration and removal of leukocytes and inflammatory debris within the inflamed area, thus facilitating the restoration of its normal physiological functions [92]. As tissue‐resident immune cells in the lung, AMs act as a central hub orchestrating both of these regulatory dimensions.

Unlike immunosuppressive anti‐inflammatory agents, proresolving mediators, including specialized proresolving mediators (SPMs–lipoxins [LXs], resolvins, protectins, maresins), proteins, and peptides, actively modulate the immune response in a tissue‐protective manner. SPMs orchestrate resolution by restricting leukocyte infiltration, recruiting and driving macrophages toward a nonphlogistic phenotype, and ultimately stimulate tissue regeneration [92, 93, 94]. SPMs exert their unique biological functions by interacting with specific cellular receptors on AMs [92]. For instance, as endogenous lipids synthesized by immune cells, particularly AMs themselves, LXs mediate anti‐inflammatory activity through G‐protein coupling to the LXA4 receptor [95].

Autocrine secretion of LXs from AMs into the lung microenvironment enhances their phagocytosis of apoptotic neutrophils [96]. LX analogs and LXA4 receptor agonists can also engage their corresponding receptors to promote the resolution of inflammation. It is reported that the LXA4 receptor agonist BML‐111 induced autophagy and inhibited apoptosis in AMs, attenuating inflammation and tissue damage by inhibiting the activation of mitogen‐activated protein kinase (MAPK)1 and MAPK8 [97]. Besides, the interaction between resolvin D2 (RvD2) and the DRV2 receptor also plays a potential proresolving role in AMs, but the precise underlying mechanisms require further investigation [98]. Collectively, these receptor–ligand interactions promote the phenotypic transition of AMs toward a proresolving state, laying the foundation for their subsequent execution of tissue clearance functions.

Infiltrated leukocytes in local tissues can be cleared via efferocytosis by AMs. Efferocytosis induces an anti‐inflammatory response featuring secretion of IL‐10 and TGF‐β, enhances clearance of apoptotic debris, promotes organ repair, and decreases antibacterial properties [99, 100, 101]. Notably, this attenuation of antibacterial capacity is not merely a phenotypic fluctuation, but rather stems from a profound metabolic reprogramming within AMs.

Intriguingly, an unbiased in vivo approach uncovered that neutrophil efferocytosis reprograms mitochondrial metabolism to switch AMs to a proresolution phenotype at the cost of bacterial control. Efferocytosis of neutrophils by AMs impaired their response to bacteria, while the uptake of epithelial cells by AMs retained their antibacterial function. Although engulfing apoptotic cells generally enhanced efferocytosis, myeloperoxidase (MPO) in neutrophil debris impaired mitochondrial ROS (mtROS) production, which is central for bacterial defense, in an uncoupling protein‐2 (UCP2)‐dependent manner. Increased UCP2 expression after neutrophil efferocytosis thus represents a conserved immunometabolic decision point that restricts the functional plasticity of TRMs, prioritizing the resolution of inflammation over host defense against bacteria [102].

In summary, AM phagocytosis is a highly plastic, context‐dependent process that requires a delicate equilibrium. Externally, their regulatory networks are easily paralyzed by viruses, manipulated by pathogens like Mtb, and inherently unequipped to process modern environmental particulates. Internally, the net benefit of reinforced resolution might come at the expense of diminished antibacterial properties of AMs, which could shift the immunological balance from bacterial containment to invasive infection [78, 103, 104]. Understanding the molecular mechanisms that regulate the balance between antibacterial and proresolving functions of AMs is clinically important. However, it remains unclear whether these opposing functions are controlled by a common upstream signaling pathway that determines the switch between them across different cellular contexts.

### 3.3 Respiratory Homeostasis Functions of AMs

#### 3.3.1 Surfactant Clearance and Metabolism

In the alveolar epithelium, AT2 cells counteract the air‐liquid interfacial tension by secreting surfactants. These surfactants safeguard gas exchange and alveolar patency [105]. In addition, approximately 40% of AMs mediate calcium signaling to alveolar epithelial cells (AECs) via gap junctions, inducing mechanical elongation of AT1 and surfactant secretion of AT2 [106]. While AT2 cells recycle a majority of the inactivated surfactants, TrAMs crucially remove the surplus of alveolar surfactants to prevent the pathological accumulation of phospholipids in the alveoli [107].

AMs internalize massive surfactant lipid–protein complexes primarily via actin‐driven macropinocytosis and targeted receptor‐mediated endocytosis. This uptake relies on a dual‐recognition system: scavenger receptors (e.g., CD36, SR‐A1) directly binding lipid moieties, alongside specific receptors recognizing SP‐A/SP‐D‐opsonized components [108]. Once internalized, these lipid cargoes undergo Rab GTPase‐regulated vesicular trafficking, sorting strictly into the late endolysosomal compartment. This rigorous membrane‐bound compartmentalization prevents cytosolic lipotoxicity while priming the lipids for subsequent enzymatic hydrolysis.

The mechanism governing this clearance depends on a paracrine signaling axis initiated by AT2 cells via the continuous secretion of GM‐CSF [109]. GM‐CSF binds to heterooligomeric receptors (composed of GM‐CSFRα and GM‐CSFRβ subunits) on the surface of AMs, triggering a transcriptional cascade essential for the establishment of the AM‐characteristic transcriptional identity and lipid‐processing capacity [110, 111]. Furthermore, GM‐CSF signaling cooperates with the transcription factor C/EBPβ to specifically induce the expression of PPAR‐γ isoform 2 [112]. PPAR‐γ subsequently promotes the expression of lipid transporters, most notably the ATP‐binding cassette (ABC) transporter ABCG1, which mediates the critical efflux of cellular cholesterol to extracellular acceptors (such as HDL), thereby maintaining intracellular lipid homeostasis [113].

Disruption at any point in this pathway abrogates the catabolic capacity of AMs, and the subsequent failure to remove surfactants results in the toxic accumulation of alveolar surface‐active substances, ultimately driving respiratory failure [110, 113]. Intracellularly, upon chronic exposure to stimuli like cigarette smoke (CS), AMs can be transformed into foamy cells with impaired surfactant clearance, characterized by severe cellular stress and mitochondrial dysfunction, directly driving airway inflammation and alveolar destruction [114]. Systemically, when GM‐CSF autoantibodies or receptor mutations abrogate this metabolic axis, the failure of AM surfactant clearance culminates in PAP, wherein massive intra‐alveolar lipoprotein accumulation precipitates severe hypoxia, respiratory failure, and opportunistic infections [108, 115].

#### 3.3.2 Efferocytosis

Distinct from the proinflammatory phagocytosis of invading pathogens, AM‐mediated efferocytosis is the targeted, anti‐inflammatory clearance of apoptotic cells [116, 117]. This specialized process is paramount for the active resolution of inflammation and the maintenance of respiratory homeostasis. This specific process is initiated by a tightly regulated “find‐me” and “eat‐me” signaling axis, predominantly through the exposure of phosphatidylserine on the apoptotic cell surface, which is recognized by specialized AM receptors such as MerTK and Axl [118, 119]. The efficient recognition and subsequent engulfment of apoptotic neutrophils and epithelial cells by AMs effectively prevent secondary necrosis, thereby halting the catastrophic leakage of damage‐associated molecular patterns (DAMPs) into the delicate alveolar space [120]. Efferocytosis extends beyond passive debris clearance to actively drive AM metabolic and transcriptional reprogramming. Specifically, intracellular degradation of apoptotic corpses shifts AMs toward a proresolving state, dampening proinflammatory responses (e.g., TNF‐α, IL‐6) while promoting the secretion of TGF‐β, IL‐10, and SPMs [99, 121]. Consequently, this AM‐driven efferocytic cascade is indispensable for the timely retraction of the inflammatory wave, ultimately restoring pulmonary structural and functional homeostasis after acute lung injuries [122, 123]. Crucially, the execution of this targeted recognition‐engulfment cascade relies on microenvironmental bridging signals.

In the alveolar niche, AT2 cell‐derived Isthmin‐1 (ISM1) stimulates adiponectin release via the GRP78/PPARγ axis. As a critical opsonin, adiponectin physically links apoptotic eosinophils to AMs. This bridging significantly lowers the engulfment threshold and ensures rapid clearance before secondary necroptosis (marked by pMLKL) occurs, effectively suppressing allergic airway inflammation [124]. After engulfment, uptake of apoptotic neutrophils introduces MPO via CD206, which upregulates UCP2. This drives a shift toward glutaminolysis, limiting mtROS accumulation. Consequently, AMs exhibit functional plasticity, sacrificing acute antibacterial defense to sustain continuous efferocytosis and prioritize tissue resolution [102].

To prevent functional exhaustion during continuous efferocytosis, TrAMs utilize the RvD1–ALX signaling axis. RvD1 binds the ALX receptor to inhibit MAPK14, thereby downregulating S100A8/A9 alarmins. This suppression enhances TrAM self‐renewal (evidenced by increased Ki67 expression) and preserves their efferocytic capacity, ensuring sustained apoptotic clearance during severe inflammation like ARDS [125].

Disruption of AM efferocytosis triggers severe systemic pathologies, recently highlighted by a lung–brain axis originating from efferocytic paralysis. High fructose‐induced metabolic stress hyperactivates the AM NLRP6 inflammasome, silencing efferocytic receptors MerTK and Tyro3. Consequently, uncleared apoptotic cells secrete serum amyloid A3 (SAA3). Platelets transport this lung‐derived SAA3 across the blood–brain barrier to the hippocampus, triggering microglial activation and neuroinflammation. Notably, this systemic pathology is reversed by the NLRP6 inhibitor mulberroside A [126]. Furthermore, preemptively calibrating AMs’ capacity for efferocytosis at the epigenetic level may also prevent such pathological outcomes. Repeated exposure to sublethal microbial stimuli induces a “trained immunity” phenotype in AMs, characterized by the robust expansion of a proresolving MERTK^hi^ subset. Mechanistically, the transcription factor Kruppel‐like factor 4 binds the Mertk promoter, epigenetically increasing chromatin accessibility for phagocytosis and lipid metabolism genes. This hardwiring accelerates apoptotic neutrophil clearance, prevents AM apoptosis, and confers tissue‐niche‐independent protection against lethal lung injury [127].

#### 3.3.3 Tissue Repair and Remodeling

Following the engulfment of apoptotic neutrophils and the subsequent resolution of acute pulmonary inflammation, AMs undergo a profound phenotypic transition from an inflammatory state to a highly specialized, proresolving phenotype. Moving beyond the traditional M1/M2 paradigm, recent scRNA‐seq reveals that extensive epigenetic and metabolic reprogramming, especially lipid and mitochondrial alterations, governs this prorepair state by driving the secretion of SPMs, IL‐10, and TGF‐β [59]. Recruited MoAMs replace the TrAMs that are largely depleted during the acute phase and drive the tissue‐remodeling response [128]. Ultimately, these AM populations coordinately promote alveolar–capillary barrier restoration and extracellular matrix (ECM) remodeling to re‐establish pulmonary homeostasis.

MoAMs regulate alveolar repair and remodeling by acting on distinct structural cells. During physiological alveolar epithelial repair, transient MoAM subpopulations supply Amphiregulin (AREG) and Wnt ligands, which promote AT2 cell proliferation and transdifferentiation into AT1 cells, thereby restoring the alveolar–capillary barrier [129]. However, during chronic fibrosis like IPF, AMs can become profoundly dysregulated. Recent spatial mapping reveals that CD206‐high MoAMs physically engage aberrant basaloid intermediates. Through spatially confined signaling networks, specifically FN‐αvβ6 and reciprocal MIF–CD74/CXCR4 interactions, these macrophages divert progenitor trajectories toward nonfunctional basal cells, driving pathological alveolar destruction [89].

Importantly, regarding ECM remodeling, MoAMs exert a potent and dual regulatory function. On one hand, they act as primary drivers of fibrogenesis by secreting classical mediators such as TGF‐β, PDGF‐A, and osteopontin (SPP1) to activate local fibroblasts for the deposition of provisional matrix proteins like fibronectin [80]. In severe conditions like rapidly progressive interstitial lung disease (RP‐ILD), MoAMs overexpress specific fibrotic genes (TGFBI, LGMN, CCL18) and release excessive chemokines (calprotectin, CCR2/CCL2 axis), establishing a positive feedback loop between inflammation and fibrosis that sustains tissue remodeling [130]. On the other hand, MoAMs are the primary source of various matrix metalloproteinases (particularly MMP‐12, MMP‐14, and MMP‐19) and cathepsins (Ctsk, Ctss). While controlled enzyme release mediates physiological ECM clearance, sustained over‐expression by MoAMs drives alveolar destruction and emphysematous remodeling [131].

### 3.4 Metabolism of AMs

Contrary to the simplistic classical view that M1 macrophages rely on glycolysis and M2 macrophages on the tricarboxylic acid (TCA) cycle [132], recent studies demonstrate that AM metabolism is flexible, context dependent, and heterogeneous, reflecting a continuous metabolic landscape rather than discrete states [61].

AMs are present in alveoli, where they are highly enriched in lipid material in the form of surfactants. AMs are critically dependent on active lipid metabolism to maintain lung surface‐active substance homeostasis and stabilize the local microenvironment [133], featuring numerous lipid droplets (LDs) within the cells and elevated expression of lipid metabolism genes such as PPAR‐γ [134]. Even under pathologic conditions, activated AMs still rely on mitochondrial respiration to meet their energy needs rather than resorting to glycolysis [135].

AMs possess a highly specialized lipid metabolism network primarily driven by GM‐CSF [112]. Besides PPAR‐γ, AMs defective in the transcription factor CCAAT/enhancer‐binding protein β (C/EBPβ) show severe defects during proliferation, phagocytosis, and lipid metabolism, revealing that C/EBPβ is a key regulator of AM cell fate and the molecular network controlling lipid metabolism in AMs [112]. This GM‐CSF‐driven transcriptional cascade upregulates scavenger receptors (such as CD36) for lipid uptake and prepares the intracellular metabolic machinery.

Following successful uptake, the internalized surfactant lipids are preferentially channeled into mitochondrial fatty acid oxidation (FAO) rather than stored. This critical metabolic shunting is heavily dependent on the rate‐limiting enzyme carnitine palmitoyl transferase 1A (CPT1A), which transports long‐chain fatty acids into mitochondria, thereby maintaining mitochondrial fitness and preventing apoptotic cell death [136]. Crucially, this FAO pathway and overall intracellular lipid processing are strictly governed by the mammalian target of rapamycin (mTOR) complex 1 (mTORC1). Mice lacking mTOR display a reduction in the number and disruption of FAO and amino acid pathways in AMs [137]. mTOR binds to the regulatory‐associated protein RAPTOR to form mTORC1, which regulates adipogenesis, and binds to the chaperone RICTOR to form mTORC2, which controls cell proliferation, growth, and cytoskeletal remodeling [138]. Deleting RAPTOR, but not RICTOR, substantially decreased AMs and induced metabolic disruptions resembling the effects observed under mTOR deficiency, confirming that mTORC1 rather than mTORC2 dictates amino acid and fatty acid metabolism in AMs [139].

Concurrently, to prevent lipotoxicity and endoplasmic reticulum stress resulting from continuous lipid engulfment, AMs also rely heavily on reverse cholesterol transport mechanisms. ABC transporters, particularly ABCG1 and ABCA1, mediate this process. Their expression is induced by liver X receptors and PPAR‐γ to promote cholesterol efflux [140, 141].

However, this delicate lipid homeostasis is highly vulnerable to various influencing factors, including aging, environmental stressors like CS, and respiratory pathogens [49]. For instance, intracellular pathogens such as *Mtb* can actively hijack AM lipid metabolism, suppressing FAO and promoting lipid re‐esterification to form nutrient‐rich LDs as a survival niche [142]. Simultaneously, oxidative stress from environmental toxins impairs mitochondrial function, forcing AMs to shunt unmetabolized lipids into extensive cytosolic LDs [143, 144]. This metabolic reprogramming transforms AMs into foam cells, with reduced phagocytic activity and increased production of lipid mediators such as prostaglandins, which are associated with chronic lung diseases including asthma and COPD [145].

Additionally, hypoxia can alter the profile of AMs metabolites and cytokine production. For example, hypoxia stabilizes hypoxia‐inducible factor (HIF)‐1α in TrAMs to promote a glycolytic phenotype, and this metabolic shift reduces their sensitivity to the inhibition of mitochondrial function, promoting cell survival during ALI [146]. However, TrAMs did not resort to enhanced glycolysis under LPS stimulation, and glycolytic inhibition had no effect on their proinflammatory cytokine production. They rely on OXPHOS to meet their energy demands and exert their functions ensured by the extremely low glucose concentrations in the microenvironment of the alveoli [135].

The glycolytic programming of AMs depends on signals from the alveolar epithelium, with distinct mechanisms operating under homeostasis and pathology. Under steady‐state surveillance, direct receptor–ligand engagement, specifically the binding of the inhibitory receptor NKR‐P1B on AMs to Clr‐g expressed on pneumocytes, is essential for maintaining metabolic homeostasis. Loss of this signaling axis induces metabolic dysregulation in AMs characterized by elevated glucose uptake and glycolytic flux [49]. Conversely, during sepsis, stressed AECs release exosomes enriched with lncRNA Rmrp to induce AM immunosuppression. Upon internalization, Rmrp stabilizes the RNA‐binding protein zinc finger protein 36 (ZFP36) by protecting it from proteasomal degradation. Consequently, ZFP36 accelerates the decay of *Pfkfb3* mRNA, a critical glycolytic enzyme. This depletion halts AM glycolytic flux, depriving them of the energy required for proinflammatory activation and exacerbating secondary pneumonia [11]. In summary, critical molecules (GM‐CSF, C/EBPβ, HIF‐1α, mTOR1) and metabolic pathways (lipid, amino acid metabolism, and glycolytic flux) regulate AM function, with metabolic balance being central to their homeostatic role.

### 3.5 Cellular Crosstalk of AMs Within the Alveolar Niche

AMs are pathogen sensors that promote immune cell recruitment to the alveoli, maintaining alveolar homeostasis and preventing the entry of immune cells into the alveolar lumen under normal conditions. By comparing the number of macrophages present in the BAL fluid and alveoli, AMs are thought to adhere to epithelial cells, and receptor proteins may be involved in facilitating this process [106]. The interaction between these key immune cells is essential for the regulation of respiratory homeostasis.

#### 3.5.1 Alveolar Epithelial Cells

AECs interact with AMs directly or via soluble mediators, which supports pulmonary homeostasis, immune tolerance, and tissue repair [147]. AT2 cells are the primary source of GM‐CSF, an indispensable growth factor that drives the differentiation of monocytes and pre‐AMs into mature AMs via the upregulation of PPAR‐γ [109]. Under steady‐state conditions, AECs maintain AM quiescence through the release of IL‐10, the activation of TGF‐β via αvβ6 integrins, and the engagement of CD200 with CD200R on AMs, thereby preventing unwarranted inflammation [76]. Moreover, during early pathogenic invasion, AT2 cells secrete SP‐A that binds to Toll‐like receptors (TLRs) on AMs to suppress NF‐κB signaling, further restraining excessive proinflammatory responses [148].

However, this suppressive environment will be breached upon substantial inflammatory challenge. Infected macrophages and monocytes produce cytokines and may activate epithelial cells to produce additional cytokines in a positive feedback loop. For instance, coculture of AMs and epithelial cells exposed to atmospheric PM10 (particulate matter) upregulates IL‐6, IL‐1β, TNF‐α, GM‐CSF, and IL‐8 expression, with synergistic increases in GM‐CSF and IL‐6 [149]. AM‐derived cytokines like IL‐1β and TNF‐α further amplify IL‐6 and IL‐8 production in epithelial cells [150, 151].

During and after lung injury, AMs deploy a diverse array of mechanisms to protect and repair the epithelial barrier. For example, during viral infections, infected epithelial and immune cells produce IFN‐I to halt viral replication, which meanwhile impedes tissue repair. In this context, AMs secrete Oncostatin M (OSM) that binds to AT2 cells and triggers robust JAK/STAT3 signaling, overriding the antiproliferative effects of IFN‐I to promote AT2 cell proliferation and barrier restoration [152]. Similarly, transitioning regenerative AMs express and release placenta‐expressed transcript 1 (Plet1), a factor that directly promotes the expansion of alveolar epithelial progenitors to replace damaged cells and facilitates the assembly of tight junction proteins to rebuild an impermeable air–blood barrier [153].

Beyond promoting AEC proliferation, AMs protect these cells from stress‐induced apoptosis. Following efferocytosis, AMs secrete resolving lipid mediators like RvD1, which induces SIRT1 expression in neighboring AECs, thereby decreasing epithelial cell apoptosis and attenuating tissue damage after lung injury [154]. Finally, this intricate crosstalk is reinforced by direct structural and vesicular communications. AMs and AECs establish connexin 43 (Cx43) gap junction channels to transmit immunosuppressive calcium waves and actively exchange exosomes carrying miRNAs that directly inhibit the NF‐κB and MAPK signaling pathways, thereby restraining excessive cytokine production and promoting the resolution of inflammation [106].

#### 3.5.2 Endothelial Cells

The precise bidirectional crosstalk between TrAMs and pulmonary microvascular endothelial cells (ECs) effectively maintains the structural and functional integrity of the alveolar–capillary barrier [155]. However, when exposure to severe pathological stressors, such as environmental toxicants or systemic infections, disrupts this delicate immune–vascular homeostasis, a cascade of intercellular pathology ensues [156].

In ALI, hyperactivated TrAMs directly induce endothelial cell death and profound vascular barrier disruption by releasing extensive proinflammatory mediators, notably IL‐1β and TNF‐α [157, 158]. Specifically, under infectious conditions, TrAMs secrete elevated levels of the peptide hormone resistin, which significantly increases endothelial permeability by downregulating essential tight junction proteins, including Claudin‐5 and occludin, through the modulation of the LKB1/AMPK/mTOR signaling axis [159].

Conversely, to mediate resolution biology, endothelial progenitor cells release miR‐218‐enriched exosomes to reprogram TrAMs, effectively suppressing their proinflammatory polarization via HMGA1 inhibition [160]. TrAM transplantation promotes a proangiogenic microenvironment through CXCL12 upregulation in endogenous AMs, which enhances EPC retention and angiogenesis, thereby accelerating alveolar capillary network restoration [161].

#### 3.5.3 Other Cells

Fibroblasts and macrophages exhibit bidirectional communication in most organs [162], influencing tissue microenvironments and disease outcomes [163]. Activated fibroblasts produce the macrophage chemotactic agent CCL2 (C–C motif chemokine ligand 2, MCP‐1), demonstrating that these cells can also attract monocytes or AMs to areas of fibrosis or injury [164]. Moreover, in severe COVID‐19 patients, AMs communicate with fibroblasts primarily through the TNFSF12–TNFRSF12A pathway, which promotes fibroblast proliferation and expression of fibrotic factors [165]. However, the precise mechanisms governing AM–fibroblast interactions in lung fibrosis remain understudied.

In addition to AT2 epithelial cells, ILC2s and basophils are other sources of GM‐CSF. AT2 epithelial cells, ILC2s, and basophils regulate AM development and function through cytokine interactions [166]. The investigators proposed that IL33 and GM‐CSF produced by AT2 epithelial cells induce further production of GM‐CSF and IL13 by ILC2s and basophils, thereby amplifying these signals [167]. This stage coincides with the period of AM development [6]. Furthermore, coculture with in vitro‐generated conditioned basophils, bone marrow‐derived macrophages (BMDMs), and immature lung macrophages may favor an AM‐like phenotype [166]. Nevertheless, the exact factors produced by pulmonary basophils that regulate AM development have not been identified. How such basophil–macrophage interactions play a role in disease and homeostatic regulation is equally worthy of investigation.

In conclusion, alveolar cells and their secreted metabolites/signal molecules form a collaborative network that enables AMs to execute immune functions and maintain lung homeostasis. Beyond the alveolar niche, this crosstalk extends to the broader airway microenvironment. Recent evidence reveals that metabolically reprogrammed airway club cells can communicate with AMs via the CXCL17–CXCR4 axis to suppress Type 2 airway inflammation [168]. This highlights that AM functionality is orchestrated by a spatially diverse network of respiratory cells, warranting further exploration of interactions between AMs and airway cells in pulmonary diseases.

## 4 Signaling Pathways in AMs

### 4.1 Hemostatic Regulating Pathways of AMs

Under physiological conditions, the establishment and long‐term residency of AMs strictly depend on continuous signals from the local alveolar niche. During early ontogeny, the GM‐CSF and TGF‐β signaling pathways instruct fetal progenitors to adapt to the lung microenvironment, terminally differentiate, and acquire their unique tissue‐resident identity [6]. Following maturation, continuous signaling through GM‐CSF, PPAR‐γ, and Wnt/β‐catenin maintains AM homeostasis. These pathways provide necessary survival signals, drive local self‐renewal, and enable basal physiological functions such as pulmonary surfactant clearance and immune tolerance (Figure 2A,B,D) [110].

**FIGURE 2:**
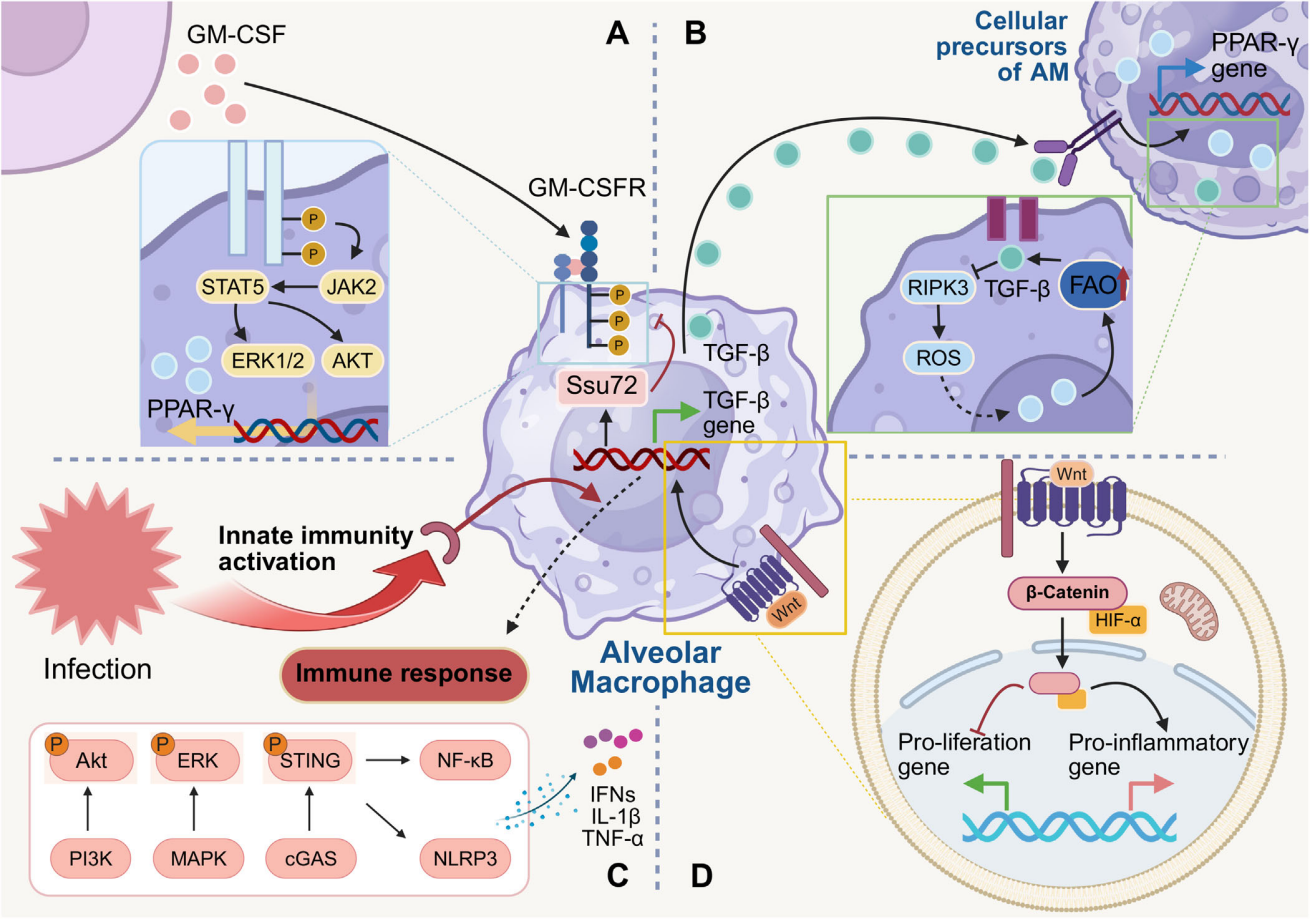
Cytokine regulation and function in AMs. (A) GM‐CSF (primarily from lung epithelial cells) binds GM‐CSFR, inducing β‐chain phosphorylation and downstream signaling (JAK2, STAT5, PKB). GM‐CSF induces PPAR‐γ transcription, while Ssu72 (upregulated by GM‐CSF) dephosphorylates the GM‐CSFR β chain to balance signaling intensity. (B) TGF‐β (autocrine/paracrine) binds its receptor, inhibiting RIPK3 to reduce ROS and activate PPAR‐γ, promoting fatty acid oxidation (FAO); FAO positively feeds back to increase TGF‐β secretion, forming a homeostatic loop. (C) Infection activates AMs, triggering innate immunity including the release of cytokines such as IFNs, IL‐1β, and TNF‐α, mediated by the PI3K/AKT, MAPK/ERK, cGAS/STING, NLRP3, and NF‐κB pathways. (D) Wnt/β‐catenin signaling balances AMs’ self‐renewal and inflammation: Wnt‐induced β‐catenin activation suppresses proliferation/stemness but promotes inflammation via β‐catenin‐HIF‐1α complex formation.

#### 4.1.1 GM‐CSF, TGF‐β, and PPAR‐γ Signaling

The GM‐CSF receptor (GM‐CSFR) comprises two chains: an α chain that directly binds GM‐CSF and a β chain critical for signal transduction [169]. Upon GM‐CSF binding to the α chain, a heterodimeric complex forms, initiating signaling through phosphorylation of the β‐chain. This is followed by the phosphorylation of downstream proteins, including activator of signal transducer and activator of transcription (STAT) 5, Janus kinase (JAK) 2, and protein kinase b (PKB) [170]. GM‐CSFR has been proven to be intimately related to the development progression of AMs. The combination of GM‐CSF with its receptors has been linked to the induction of the transcription factor PPAR‐γ and the corresponding initiation of AMs differentiation in fetal lung mononuclear cells during prenatal development [171, 172]. For instance, intranasal administration of exogenous GM‐CSF has been shown to restore AM populations in mice lacking GM‐CSF, suggesting a crucial role for GM‐CSF in the evolution of AMs [6].

AMs originate from fetal monocytes and undergo differentiation into long‐lived cells within the first week of life, a process dependent on GM‐CSF [6]. Intensive studies have shown that the transition from pre‐AMs to AMs necessitates the upregulation of PPAR‐γ, which is stimulated by exposure to GM‐CSF [110]. This result further elucidates the mechanisms by which the GM‐CSFR signaling axis is involved in AM growth and development. However, evidence from various tissues under inflammatory conditions indicates that GM‐CSF may also be produced by other sources, including natural killer cells, ILCs, T cells, B cells, endothelial cells, and fibroblasts [173]. The primary cellular source of GM‐CSF that governs AMs differentiation remains an open question.

AMs reciprocally regulate GM‐CSFR signaling intensity. Upon treatment with GM‐CSF, AMs promptly increase the expression of the RNA polymerase II subunit A C‐terminal domain phosphatase SSU72 (Ssu72). This enzyme degrades the intensity of GM‐CSFR signaling by combining directly with the GM‐CSFR β chain and dephosphorylating it. In cells lacking Ssu72, the GM‐CSFR β chain, and its downstream signaling proteins are hyperphosphorylated, leading to developmental abnormalities in AMs and numerous disruptions in cell number, the cell cycle, proliferation, death, and so on. JAK2 inhibitors reverse these abnormalities, highlighting a critical negative feedback balance between GM‐CSF signaling and Ssu72‐mediated regulation that is essential for AM homeostasis [174].

TGF‐β is a multifunctional cytokine that belongs to the TGF superfamily. It becomes activated and binds to the TGF‐β receptor, which comprises Type 1 and Type 2 receptor subunits. Following binding to TGF‐β, TGF‐β Type II2 receptor (TGFBR2) kinases phosphorylate and initiate the signaling cascade [175]. This process activates various downstream substrates and regulatory proteins, leading to the transcription of different target genes that are involved in the differentiation, chemotaxis, proliferation, and activation of various immune cells [176, 177].

AMs are the main source of TGF‐β [178]. During the recovery phase of lung injury, AMs release TGF‐β in an autocrine manner. TGF‐β induces SMAD2/3 phosphorylation in both AMs and monocyte‑derived macrophages (MDMs). By interacting with non‐Wnt pathways (mainly relying on the high expression of TGFBR2), TGF‐β significantly increases Ki67 expression in AMs, driving their proliferation and self‐maintenance. Meanwhile, AM‐derived TGF‐β significantly inhibits MDM proliferation. In MDMs, key Wnt pathway genes (such as *Ctnnb1* and *Lrp5/6*) are upregulated, while Ki67 and cell cycle‐related genes are downregulated. Moreover, TGF‐β shifts MDMs from an inflammatory phenotype (Ly6C^hi^, inducible nitric oxide synthase [iNOS^+^]) to an anti‐inflammatory phenotype (CD206^+^). MDMs highly express the Wnt coreceptors LRP5/6, whereas AMs highly express the TGFBR2, and this difference in receptor expression determines that TGF‐β triggers different transcriptional programs in the two cell types [179]. AMs secrete TGF‐β and sense this signal through the receptors mentioned above, forming an autocrine positive loop.

TGF‐β is also crucial for AM development [46]. It drives CCR2^+^ monocyte differentiation into MoAMs, linking monocyte recruitment to alloimmunity and bronchiolitis obliterans syndrome [180]. AMs can produce a range of cytokines that are necessary in both the injury and repair phases of acute lung inflammation. Specifically, IL‐6 is associated primarily with the injury phase, whereas TGF‐β is key in the subsequent repair process. These findings indicate that in the context of acute complications following lung transplantation, AMs undergo early activation concurrently with excessive production of IL‐6. This cytokine plays a role in tissue injury and is later countered by a phase of activation characterized by the predominance of TGF‐β, which facilitates tissue repair [181]. Efferocytosis promotes AMs releasing anti‐inflammatory factors, including platelet‐activating factor, TGF‐β, and PGE2, which in turn inhibits inflammation [99, 182]. AMs support the production of regulatory T cells by secreting critical factors such as retinal dehydrogenases 1 and 2 and TGF‐β [183].

Maintaining homeostasis requires continuous TGF‐β signaling in AMs, which is linked to PPAR‐γ. Mechanistically, TGF‐β signaling triggers the upregulation of PPAR‐γ, a critical transcription factor required for the development of AMs. PPAR plays a crucial role in regulating cell differentiation, development, metabolism, and tumorigenesis in higher organisms [184, 185, 186]. Compared with other macrophage populations, AMs exhibit a unique expression pattern of genes involved in lipid metabolism [187]. Notably, mice that lack PPAR‐γ expression in AMs exhibit spontaneous inflammation in the lungs, highlighting the crucial role of AM lipid metabolism in maintaining airway homeostasis [110].

Furthermore, PPARγ is the master regulator which helps maintain the homeostatic identity genes that define TrAMs, including *SiglecF*, *Marco*, and* Ear1*. Under Type 2 inflammatory conditions, however, this PPARγ‐dependent network is dismantled. Allergens like house dust mite (HDM) or IL‐33 stimulate ILC2s to release IL‐13, which signals through IL‐4Rα on TrAMs and strongly induces the transcription factor IRF4. IRF4 then binds directly to the *Pparg* locus and represses PPARγ transcription, which leads to the downregulation of steady‐state signature genes related to TrAM identity. Also, IRF4 interacts with other sets of gene expression programs, namely, the chemotaxis regulon and the cell‐fusion regulon. IRF4 induces chemokines such as CCL24, which actively recruit inflammatory cells like eosinophils, and induces a series of cell‐fusion‐related genes, including *Ocstamp*, *Dcstamp*, *Kcnn4*, *Plekho1*, and *Adam8*, driving the formation of multinucleated giant cells. As a result, TrAMs are repurposed toward the proinflammatory phenotype [62].

In‐depth studies have revealed that complex interconnecting mechanisms exist between functional regulators in AM growth and development. For example, targeted removal of TGF‐β receptor 2 in the adult mouse AMs resulted in disruption of AM homeostasis and reduced PPAR‐γ expression. Furthermore, GM‐CSF increased PPAR‐γ expression and functional activity [110]. PPAR‐γ expression is absent in PAP patients and GM‐CSF‐deficient mice. In patients and mice lacking GM‐CSF, AMs exhibited excessive lipid accumulation. However, overexpressing PPAR‐γ in airway macrophages of GM‐CSF‐deficient mice restored their ability to clear lipids [188]. Despite these insights, the interplay between TGF‐β and PPAR‐γ likely involves additional cofactors or epigenetic modifications, which have not yet been fully characterized and need further studies.

#### 4.1.2 Wnt/β‐Catenin Signaling Pathway

The Wnt/β‐catenin signaling pathway serves as the central regulator of AM self‐renewal and inflammatory responses [189, 190]. Under homeostatic conditions, this canonical pathway maintains the highly stable AM pool, where over 90% of the population is sustained through local GM‐CSF‐dependent proliferation rather than circulating monocyte recruitment [6]. Mechanistically, Wnt ligands bind to Frizzled receptors at baseline, preventing β‐catenin degradation and promoting its nuclear accumulation. This mechanism effectively preserves the stemness of the principal self‐renewing population in the alveolar niche, known as proliferating AMs (characterized by Ki67^+^ expression). However, upon immune activation, the Wnt/β‐catenin pathway exerts a paradoxical, dual regulatory effect: it suppresses AM self‐renewal while simultaneously enhancing proinflammatory functions [190, 191]. This phenotypic switch is driven by profound metabolic reprogramming. Specifically, β‐catenin interacts with HIF‐1α to form a complex that promotes a glycolytic shift, thereby fueling inflammatory responses. Conversely, this β‐catenin/HIF‐1α complex actively suppresses the mitochondrial metabolism required to sustain AM proliferation both in vivo and in vitro [69, 190, 192, 193]. During acute lung inflammation, AMs depleted by pathogen phagocytosis are rapidly replenished through a combination of monocyte influx and accelerated local proliferation [194].

Following the resolution of inflammation, the Wnt‐driven self‐renewal program re‐establishes long‐term population stability [194]. Moreover, MDMs infiltrate extensively, and a subset differentiates into MoAMs. Single‑cell transcriptomics has identified a transitional monocyte‑derived subset, termed Mac0, which precedes mature AM formation. Both Notch and Wnt/β‑catenin pathways are enriched in Mac0 and AM clusters. Notch/RBP‑J signaling promotes the transition of monocyte‑derived IMs into Mac0 cells that acquire AM‑like features, and Wnt signaling acts at a later stage, driving Mac0 toward fully differentiated, functional AMs.

The downstream effector of Wnt/β‑catenin (encoded by *Ctnnb1*) is critical for proper differentiation. Monocyte‑specific deletion of *Ctnnb1* derails the MDM differentiation trajectory, favoring premature or incomplete conversion of MoIMs to MoAMs. These β‑catenin‑deficient MoAMs exhibit impaired phagocytosis and elevated expression of profibrotic genes. Thus, the sequential engagement of Notch and Wnt signals coordinates the stepwise maturation of MDMs into AMs, and the timely action of Wnt/β‑catenin is essential for generating competent, tissue‑resident‑like AMs that restrain fibrotic remodelling [53]. Notably, certain pathogens can exploit these finely tuned dynamics. During *Mtb* infection, for instance, proliferation is restricted entirely to uninfected AMs. Infected cells instead undergo cell‐cycle arrest, a phenomenon evidenced by the mutual exclusivity of Ki67 and iNOS expression, suggesting that *Mtb* hijacks this Wnt‐associated metabolic pathway as a mechanism of immune evasion [61, 195, 196].

### 4.2 Disease‐Associated Signaling Pathways

Unlike the hemostatic pathways that maintain the development of AMs, disease‐associated signaling cascades are stress‐responsive. Upon pathogen exposure or injury, pathways like NF‐κB, JAK/STAT, and NLRP3 are transiently activated, enabling AMs to execute acute inflammatory clearance and subsequently drive tissue resolution [197]. However, persistent activation of these pathways by chronic stimuli shifts AMs toward a dysfunctional phenotype, driving the continuous release of inflammatory and profibrotic mediators that promote severe pulmonary diseases like ALI and IPF [5]. The specific mechanisms of these major stress‐responsive pathways are detailed below (Figure 2C).

#### 4.2.1 IFN Signaling

IFNs constitute a family of signaling proteins that are produced and secreted by host cells. These molecules serve as a means of intercellular communication, initiating the immune system's protective defenses and aiding in the elimination of pathogens. They are categorized into three types: Type I (IFN‐α/β), Type II (IFN‐γ), and Type III (IFN‐λ). IFNs also exhibit a range of functions, including the activation of immune cells such as macrophages and natural killer cells. They enhance the host's defense mechanisms by increasing antigen presentation, which is achieved through increased expression of MHC antigens [198]. Each IFN subtype exhibits distinct functions and signaling pathways, which are critical for shaping immune responses to pathogens and maintaining respiratory homeostasis.

The functional roles of IFN subtypes in AM regulation are distinct. IFN‐γ dominates disease development and is associated with increased TNF‐α production and AM depletion. Conversely, influenza‐induced IFN‐γ decreases AM‐mediated sterilization, thus expanding the predisposition of the host to pneumococcal infection [199]. IFN‐γ signaling also impairs αβ T‐cell recruitment and AM survival during influenza development [200]. In contrast to IFN‐γ, IFN‐β in vitro reversed AMs cytokine‐induced TNF‐α/IL‐10‐mediated injury, recovered alveolar innate immune responses, and enhanced bacterial clearance and recruitment of alveolar neutrophils. Systemic administration of IFN‐β enhances survival by restoring the impaired function of AMs, primarily through the recruitment of neutrophils to the alveoli [201]. In influenza‐induced *S. pneumoniae* coinfections, Type I IFNs (IFN‐α/β) suppress early viral replication and inflammation [202, 203]. However, IFN‐γ’s role in inhibiting AM function complicates this balance. The interplay between Type I and Type II IFNs highlights their dual roles in immunity: Type I IFNs control viral infections, while Type II IFNs may exacerbate inflammation. Further research is needed to clarify their specific functions and interactions in AM regulation.

TrAMs represent a critical immune cell subset in the pulmonary microenvironment that specifically expresses the IFN‐λ receptor (IFNLR1) and robustly responds to this cytokine [204]. During respiratory pathogen invasion, IFN‐λ engages the IFNLR1/IL10Rβ receptors on AMs to activate JAK–STAT signaling and robust interferon‐stimulated genes (ISGs) expression, thereby orchestrating a localized antiviral state that suppresses viral replication without causing collateral tissue damage [204, 205]. Furthermore, in acute inflammatory events, IFN‐λ attenuates proinflammatory polarization by downregulating NF‐κB activation, and reduces oxidative stress by promoting Nrf2‐mediated antioxidant pathways, ultimately preventing uncontrolled alveolar inflammation [206].

#### 4.2.2 The NLRP3 Inflammasome Pathway

TrAMs are strictly adapted to a unique high‐oxygen, lipid‐rich microenvironment, serving as the primary innate sensors for pulmonary inflammation [76]. In AMs, ligation of TLR4 by LPS initiates NF‐κB signaling, upregulating the transcription of NLRP3, pro‐IL‐1β, and pro‐IL‐18. Following secondary chemical signals, such as K^+^ efflux induced by nigericin or extracellular ATP, mitochondrial swelling and cardiolipin externalization occur, which activate NLRP3 [207]. Alternatively, physical stressors like irradiation directly promote NLRP3 binding to ASC, bypassing canonical NF‐κB signaling to trigger activation [208]. Upon activation, NLRP3 recruits ASC and Procaspase‐1 to assemble the inflammasome complex, facilitating Procaspase‐1 autocleavage. Active Caspase‐1 processes pro‐ILs into their mature forms and cleaves gasdermin D (GSDMD). The N‐terminal GSDMD fragment executes pyroptosis by disrupting membrane integrity, thereby releasing IL‐1β, IL‐18, and DAMPs (e.g., ATP, HMGB1) into the alveolar space. This efflux establishes a feed‐forward inflammatory loop: it recruits neutrophils, whose ROS and neutrophil extracellular traps (NETs) further amplify local damage and reinforce NLRP3 activation. Secreted TNF‐α and IL‐6 simultaneously act in an autocrine/paracrine manner to drive a proinflammatory (CD206^−^) AM phenotype, exacerbating tissue injury and subsequent fibrosis [207, 208].

Beyond these classical receptor‐mediated cascades, the low‐glucose, high‐oxygen alveolar niche confers OXPHOS dependency, and itaconate acts as a tissue‐specific metabolic regulator of NLRP3 activation in AMs. TLR4 activation strongly induces IRG1 expression, catalyzing the conversion of cis‐aconitate to itaconate. LPS‐stimulated AMs produce approximately twofold more itaconate than BMDMs, and elevated itaconate levels in the BALF of pneumonia patients confirm its clinical presence [209]. Mechanistically, independent of NRF2 and GSDMD signaling, native itaconate competitively inhibits succinate dehydrogenase (SDH), causing intracellular succinate accumulation and an elevated succinate‐to‐fumarate ratio. To compensate for SDH inhibition, AMs upregulate mitochondrial electron transport chain complex I (ETC‐CI) activity. This metabolic adaptation increases the oxygen consumption rate and intracellular ATP levels, directly triggering NLRP3 inflammasome assembly. This specific metabolic dependency is rigidly governed by the alveolar microenvironment. AMs lose their proinflammatory responsiveness to itaconate after 3 days of ex vivo culture, whereas BMDMs transplanted into the alveolar space acquire this phenotype [209].

#### 4.2.3 The PI3K/AKT Pathway

The PI3K/AKT cascade regulates the cell cycle, proliferation, cellular metabolism, and M2 macrophage polarization. In AMs, PI3K/AKT signaling closely couples amino acid availability to inflammasome activation. Under inflammatory conditions, NF‐κB and JAK/STAT1 signaling upregulates the glycine transporter SLC6A9 to maintain intracellular glycine levels, which sustains PI3K/AKT1 and downstream mTOR activation. This cascade facilitates NLRP3 inflammasome assembly and Caspase‐1‐mediated IL‐1β maturation. Conversely, SLC6A9 inhibition causes extracellular glycine accumulation. Extracellular glycine engages the receptor Glra4, suppressing PI3K activity and reducing AKT1 phosphorylation. This targeted suppression dampens mTOR‐dependent IL‐1β production without altering general inflammatory transcripts such as TNF‐α or IL‐6 [210].

Beyond metabolic coupling, the PI3K/AKT/mTOR axis operates as a classical negative regulator of autophagy. In sepsis‐induced ALI, sustained PI3K/AKT activation impairs autophagosome formation in AMs, causing damaged mitochondria to accumulate. The resulting release of mitochondrial ROS and DNA triggers NLRP3/Caspase‐1/GSDMD‐mediated pyroptosis. Andrographolide by binding the RAGE receptor inhibits the PI3K/AKT/mTOR cascade, restoring LC3 lipidation (LC3‐I to LC3‐II) and autophagosome formation. Restored autophagy restricts inflammasome hyperactivation by clearing mitochondrial DAMPs and degrading inflammasome components [211].

AMs can also activate AKT independently of PI3K under severe environmental stress. Specifically, silica‑induced ROS promote the oligomerization of peroxiredoxin 4 (PRDX4). The oligomeric form of PRDX4 oxidizes PTEN monomers at Cys124 and Cys245, disrupting PTEN homodimer formation and thereby relieving PTEN‐mediated suppression of AKT Thr308 phosphorylation. This PI3K‐independent axis sustains robust NF‐κB nuclear translocation, driving the continuous expression of proinflammatory and profibrotic cytokines, including TNF‐α, IL‐1β, IL‐6, and TGF‐β [212].

#### 4.2.4 The NF‐κB Pathway

The NF‐κB signaling pathway serves as a central regulator of inflammatory responses in AMs [213, 214]. Under physiological conditions, NF‐κB is sequestered in the cytoplasm by IκB inhibitors. Upon stimulation, IKK phosphorylates IκB, leading to its ubiquitination and degradation. This releases the p65 subunit, allowing it to translocate to the nucleus and initiate the transcription of proinflammatory cytokines [215, 216].

During viral infections, pathogens frequently hijack distinct upstream sensors to hyperactivate NF‐κB in AMs. For instance, ASFV activates the AKT/mTOR axis, driving excessive NF‐κB‐mediated cytokine production in porcine AMs [217]. Beyond exogenous pathogens, intrinsic transcriptomic and metabolic shifts also converge on this pathway to amplify inflammation. Epigenetically, the circular RNA circ‐Phkb acts as a positive regulator that sensitizes AMs to inflammatory apoptosis by upregulating the upstream TLR4/MyD88 cascade [218]. Concurrently, metabolic stress conditions, such as the combination of low glucose and elevated β‐hydroxybutyrate, exacerbate AM inflammation by activating the GPR109A/p38 signaling axis, which subsequently phosphorylates NF‐κB [219]. These findings indicate that infectious, transcriptomic, and metabolic signals collectively utilize the NF‐κB pathway to shape the proinflammatory phenotype of AMs.

Endogenous mechanisms that restrain this pathway are also critical for immune homeostasis. The mechanosensitive channel transient receptor potential vanilloid 4 directly binds to p65 at the endoplasmic reticulum to constrain excessive inflammatory responses. This physical interaction prevents IκBα degradation and restricts p65 nuclear translocation, thereby limiting inflammatory damage caused by NF‐κB [220].

#### 4.2.5 The MAPK Pathway

The MAPK signaling pathway is a central mechanism mediating responses to environmental stress and pathogens [221, 222]. This cascade comprises three main branches including extracellular signal‐regulated kinase (ERK), c‐Jun N‐terminal kinase (JNK), and p38 MAPK. Upon receptor activation, sequential phosphorylation of these kinases drives transcription factor activation and subsequent proinflammatory cytokine production [221, 222]. In the respiratory system, AMs utilize MAPK signaling to execute innate defense mechanisms, while excessive activation of this pathway contributes to severe respiratory conditions, such as ALI [125, 223].

Pathogen‐derived components typically trigger these pathways. For instance, lipoteichoic acid (LTA) from Gram‐positive bacteria is a recognized MAPK activator [224]. In murine AMs, LTA stimulation induces rapid phosphorylation of JNK and p38 MAPK, leading to elevated expression of IL‐1β and iNOS [225]. During viral infections, porcine reproductive and respiratory syndrome virus (PRRSV) exploits this mechanism by inducing a delayed p38 MAPK activation in primary porcine alveolar macrophages (PAMs) at late infection stages, which facilitates viral replication. Counteracting this viral strategy, treatment with the isoquinoline alkaloid lycorine hydrochloride triggers an early and rapid increase in p‐p38 levels in PAMs, independent of JNK and ERK activities [226]. This early p38 boosting promptly activates the downstream NLRP3/Caspase‐1/IL‐1β cascade, establishing an early antiviral state that suppresses PRRSV proliferation. These findings suggest that both the specific branch targeted and the temporal sequence of activation within the MAPK network dictate the immunological responses of AMs.

#### 4.2.6 The JAK/STAT Pathway

The JAK/STAT signaling pathway is a primary regulatory cascade for immune and inflammatory responses in AMs [227]. Upon pathogen recognition, IFNs bind to cell surface receptors, initiating the phosphorylation of JAKs. This event leads to the activation and nuclear translocation of STAT proteins, which regulate the transcription of numerous downstream genes, including ISGs and proinflammatory cytokines [228, 229]. In the alveolar microenvironment, the modulation of JAK and STAT isoforms in AMs influences the balance between viral clearance and tissue injury.

During viral infections, robust activation of the JAK/STAT axis in AMs is required to restrict pathogen replication. In PAMs, viruses such as the PRRSV often evade innate immunity by suppressing IFN‐mediated signaling [230, 231]. Dicoumarol‐graphene oxide quantum dots (DIC‐GQDs) exert anti‐PRRSV effects by inducing the phosphorylation of TYK2, JAK1, STAT1, and STAT2, resulting in the upregulation of IFN‐β and subsequent antiviral ISGs [232]. Exposure to severe environmental stress can trigger hyperactivation of specific JAK/STAT branches, leading to ALI and pulmonary fibrosis. Zymosan, a yeast cell wall derivative, selectively induces the rapid phosphorylation of JAK1 and STAT3 in AMs. This specific STAT3 activation drives the extensive production of proinflammatory mediators, including IL‐6, TNF‐α, and iNOS [233].

#### 4.2.7 The cGAS–STING Pathway

The cGAS–STING pathway is a primary innate immune sensing mechanism that detects cytosolic double‐stranded DNA [234]. Upon binding DNA, cGAS synthesizes the second messenger cGAMP, which binds to STING on the endoplasmic reticulum [235]. STING subsequently translocates to the Golgi apparatus, recruiting TANK‐binding kinase 1 to phosphorylate interferon regulatory factor 3 (IRF3), thereby driving the transcription of Type I IFNs and inflammatory cytokines [234, 235]. In the pulmonary microenvironment, AMs utilize this signaling axis to respond to cellular damage and lung inflammation [236].

Mitochondrial DNA (mtDNA) is an endogenous trigger for cGAS–STING activation within AMs during sterile inflammatory diseases. In ARDS, stimulation by LPS induces severe mitochondrial dysfunction in AMs, characterized by membrane potential collapse and ROS overproduction. This oxidative stress opens the mitochondrial permeability transition pore, allowing mtDNA to escape into the cytosol and activate the cGAS–STING–IRF3 cascade [236]. STING activation phosphorylates both IRF3 and IRF7, which creates a feed‐forward loop that significantly lowers the activation threshold for the NLRP3 inflammasome [237]. Consequently, the cGAS–STING–IRF3/IRF7 axis drives massive AM pyroptosis and the release of IL‐1β into the alveolar space. Pharmacological interventions targeting this upstream process, such as 4‐octyl itaconate, restore mitochondrial homeostasis in AMs, downregulate cGAS and STING protein expression, and prevent pyroptotic lung injury [236].

AMs regulate pulmonary inflammation by modulating cGAS–STING activity in the surrounding alveolar microenvironment. During bleomycin‐induced lung injury, profibrotic AMs actively secrete exosomes enriched with the pattern‐recognition molecule Ficolin B (Fcn B). Upon uptake by lung epithelial cells, these AM‐derived exosomes deliver Fcn B into the cytosol to trigger the epithelial cGAS–STING pathway. Instead of typical IFN production, this specific STING activation induces ferroptosis of the epithelial barrier [238].

## 5 AMs in Pulmonary Diseases

AMs are central to the balance between immune defense and tissue damage in respiratory diseases [76], with dysregulation of their subsets, phenotypes, and metabolism driving pathology. Inflammatory responses are aimed at clearing pathogens but often inevitably lead to tissue damage. Beyond the classical M1/M2 paradigm, recent studies highlight diverse AM subtypes with distinct functions in disease [239, 240], which are integrated below where relevant (Table 1).

**TABLE 1:** The role of AMs in pulmonary diseases.

Pulmonary diseases	The traits of AMs in diseases	Mechanisms	The role of AMs in diseases	References
**Pneumonia (bacterial)**				
Acute phase	Increased CD11c^+^ CD11b^+^ AMs; Impaired chemotactic function	6PPD‐Q respiratory exposure → substantially increases CD11c^+^ CD11b^+^ AM populations → reduces expression of CCL2, CXCL1, and CXCL2 → impairs AM chemotactic function	Exacerbates the severity of *Klebsiella pneumoniae* pneumonia	[241]
Postresolution phase	Prolonged paralyzed state; poor phagocytosis; immune tolerance	Postsepsis AT2 cells release Rmrp‐enriched exosomes → AMs internalize Rmrp → stabilizes ZFP36 → targeted decay of Pfkfb3 mRNA → depletes PFKFB3 → severely impairs aerobic glycolysis	Forces AMs into immune tolerance; renders lung highly vulnerable to secondary bacterial pneumonia	[11]
**Pneumonia (viral)**				
Acute phase	Peroxisome deficiency; lipid metabolic dysfunction	Viral infection (e.g., SARS‐CoV‐2) → peroxisome deficiency and lipid metabolic dysfunction → fails to produce antiviral IFNs → overproduces proinflammatory cytokines (IL‐1β via ROS‐mediated inflammasome activation)	Exacerbates ALI; impedes early epithelial defense	[242, 243]
Resolution phase	Tissue‐regenerative phenotype	IAV‐induced depletion of TrAMs → recruited BMDMs undergo spatiotemporal transition → upregulate and shed Plet1 → soluble Plet1 activates Raf–MEK–ERK and suppresses Src kinase in epithelial cells → drives epithelial progenitor proliferation and upregulates tight junction proteins	Actively promotes resealing of the epithelial barrier and restoration of pulmonary homeostasis	[153]
Postrecovery	Profound epigenetic reprogramming; innate immune memory; downregulated fatty acid metabolism	Shifts chromatin accessibility toward IFN‐related pathways (STAT1/2) and restricts proinflammatory NF‐κB → mounts a highly balanced response upon secondary viral challenge	Enhances secondary antiviral defense and reduces lung tissue damage	[244]
**Asthma**				
Homeostasis	Restrictive/protective phenotype; upregulated MHC II and CD11b (if TRIM33 deleted)	TRIM33 binds to the Ccl2 promoter in AMs → suppresses CCL2 transcription (specific deletion of TRIM33 → CCL2 surge → influx of inflammatory CD11b^+^ dendritic cells → amplifies Th2 priming and cytokines)	Central regulators suppressing allergic responses; prevents asthma exacerbation	[245]
Asthmatic microenvironment	Proinflammatory drivers	IgE stimulation → releases cytokines (IL‐1, TNF‐α, IL‐6, GM‐CSF) and histamine‐releasing factors/releases cysteinyl leukotrienes (LTC4, LTD4, LTE4) → prolongs eosinophil survival, sustains mast cell/basophil activation, and induces mucus hypersecretion	Exacerbates airway pathology	[246, 247, 248, 249]
Virus‐induced exacerbation	Impaired antiviral capacity	TSLP‐driven upregulation of CUL5 in TrAMs → proteasomal degradation of OGT → loss of MAVS O‐GlcNAcylation → blocks RIG‐I antiviral signaling and IFN‐β production → uncontrolled viral replication	Drives neutrophilic asthma exacerbations	[250]
**COPD**				
Metabolic exhaustion	Metabolic exhaustion; phagocytosis impairment	CS exposure → suppresses SIRT1/MKP‐1 axis → hyperactivates MAPK and NF‐κB pathways/downregulation of NRF2‐regulated ME1 → disrupts redox balance and depletes TCA cycle intermediates → over‐reliance on glycolysis	Deprives AMs of energy and antioxidant capacity; fails to clear pathogens/apoptotic cells	[14, 251]
Dyslipidemia	Proinflammatory M1 phenotype; paralyzed phagocytosis	Aberrant accumulation of cholesterol → blunts mitochondrial respiration and massive ROS leakage → activates PPIA/NF‐κB cascade → secretes excessive IL‐1β, TNF‐α, IL‐6/NF‐κB‐mediated CD206 downregulation and ATP depletion	Structurally and energetically paralyzes phagocytosis	[252]
Apoptosis resistance	Apoptosis resistant; hyperactive and hyper‐secretory state	Dysregulation of PPAR‐γ and compromised ISM1/csGRP78‐mediated apoptosis → bypasses normal cellular senescence (p21 downregulation) → unleashes unabated proteolytic assault (MMP‐12)	Perpetuates persistent airway inflammation; drives irreversible ECM degradation and emphysema	[253, 254]
**CF**				
Phagocytosis defect	CFTR‐deficient AMs (predominantly MoAMs); Defective phagolysosomal acidification; impaired pathogen clearance	CFTR mutation/dysfunction in AMs → fails to regulate pH and acidify lysosomes → inability to phagocytose lysosomal vesicles → bacterial clearance defect → survival of opportunistic pathogens (*S. aureus*, *P. aeruginosa*)	Perpetuates chronic bacterial infections	[255, 256]
Hyper‐inflammation	Hyper‐inflammatory phenotype; increased secretion of proinflammatory cytokines (IL‐6, IL‐8)	Continuous inflammatory stimulation → upregulates XBP1 and downregulates Type I IFN signaling in CFTR‐mutated Ams → excessive secretion of IL‐6 and IL‐8	Induces intense airway inflammation; causes lung injury	[257, 258, 259]
**IPF**				
External reciprocal signaling	Profibrotic MoAMs	Fibroblast‐derived CXCL12 binds to CXCR4 on MoAMs → triggers activation of the MEK/ERK pathway → induces MoAM chemotaxis and alters reparative properties	Drives fibrotic progression; exacerbates IPF	[260]
Internal transcriptional networks	Upregulated TREM2, SPP1, and APOE; downregulated PPARG	Sphingomyelin activates elevated TREM2 to protect MoAMs from apoptosis and downregulation of PPARG removes negative regulation of SPP1 → SPP1 interacts with CD44 → secretes profibrotic mediators like MMP12 and promotes EMT pathway	Amplifies profibrotic signaling; inhibits regeneration of AT2 cells	[64, 261, 262]
Lung cancer (adenocarcinoma)	M2 polarization; reduced CD40/80/86; increased CXCL1, CXCL2, IL‐1	Inflammatory tumor microenvironment (e.g., mutant EGFR) → induces AM transition to TAMs with M2 polarization → reduces CD40/80/86 and increases tumor‐promoting chemokines (CXCL1, CXCL2, IL‐1)	Directly drives tumor progression	[263]
**Lung cancer (AAH)**				
Precancerous stage	Proangiogenic signature; S100a4^+^ AMs	Enhanced CPT1A‐mediated fatty acid oxidation → metabolic shift confers proangiogenic signature (elevated ANXA2, VEGF, HIF‐1α)	Drives endothelial tube formation and epithelial proliferation	[141]
**Lung cancer (NSCLC)**				
Early tumorigenesis	SASP; p16^+^ CXCR1^+^	TrAMs develop SASP (elevated p16^INK4a^, CXCR1, SA‐β‐Gal) → release factors that suppress the infiltration and expansion of cytotoxic CD8^+^ T cells	Orchestrates an immunosuppressive TME; drives immune evasion	[264]
Preinvasive stage	Protumorigenic phenotype	AT2 cells secrete AREG → activates EGFR in fibroblasts → produce TNC → TNC acts on AMs via TLR4 → induces tumor‐promoting state (high Msr1, low MHC II)	Impairs neutrophil and γδ T cell recruitment; constructs a permissive oncogenic niche	[265]

### 5.1 Pneumonia

Pneumonia, an acute pulmonary inflammation typically triggered by viral or bacterial infections, is intricately governed by the host's innate immune response [266, 267]. As the primary resident immune cells, AMs interact bidirectionally with pneumonia, critically influencing disease progression while in turn being reshaped by its phases (Figure 3A) [268]. This airway environment favors the transition of pathogenic bacteria such as *Pseudomonas aeruginosa* (*P. aeruginosa*) from acute virulence to chronic persistence [269].

**FIGURE 3:**
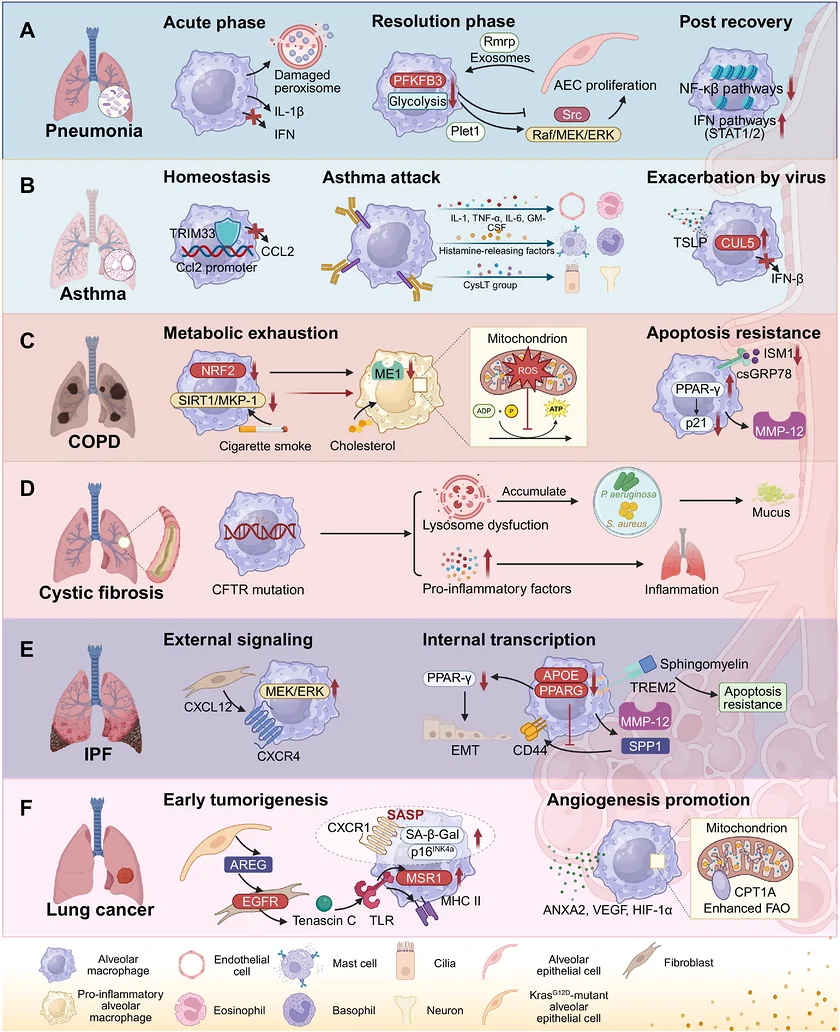
Functions of AMs in diverse pulmonary diseases. (A) Pneumonia: During the acute phase, AMs are susceptible to environmental stressors and virus‐induced peroxisome deficiency, leading to impaired chemotactic function and hyperinflammation (increased IL‐1β, decreased IFN). In the resolution phase, AT2 cell‐derived exosomes containing lncRNA Rmrp stabilize ZFP36 to degrade Pfkfb3 mRNA, inhibiting glycolysis in AMs. Also, recruited AMs shed Plet1 to activate Raf–MEK–ERK signaling and suppress Src, promoting epithelial repair. After recovery, memory AMs shift chromatin accessibility toward IFN pathways and restrict NF‐κB for protective innate immune memory. (B) Asthma: Under homeostasis, TRIM33 binds the Ccl2 promoter to suppress its transcription. During an asthma attack, IgE‐activated AMs release proinflammatory cytokines and lipid mediators (CysLTs) to amplify inflammation. In virus‐induced exacerbation, TSLP‐driven upregulation of CUL5 triggers OGT degradation, blocking RIG‐I antiviral signaling and IFN‐β production. (C) COPD: CS and cholesterol overload suppress the SIRT1/MKP‐1 axis and downregulate NRF2‐regulated ME1, causing metabolic exhaustion with impaired OXPHOS and excessive ROS. Dysfunctional AMs evade clearance via PPAR‐γ dysregulation and compromised ISM1/csGRP78‐mediated apoptosis, adopting an apoptosis‐resistant state and releasing MMP‐12 to drive emphysema. (D) Cystic fibrosis: CFTR mutations in AMs cause lysosome dysfunction, impairing phagosome–lysosome fusion and lysosomal acidification. This bacterial clearance defect leads to the intracellular survival and accumulation of S. aureus and P. aeruginosa. CFTR dysfunction also drives a hyperinflammatory phenotype with increased proinflammatory cytokine secretion. (E) IPF: MoAMs are primary drivers. Externally, fibroblast‐derived CXCL12 binds CXCR4 on MoAMs to activate MEK/ERK signaling, inducing chemotaxis. Internally, MoAMs downregulate PPARG, allowing SPP1 to interact with CD44 and promote fibrosis via EMT. Alveolar sphingomyelin activates TREM2, conferring a survival advantage and driving a profibrotic phenotype that secretes MMP‐12 and SPP1. (F) Lung cancer: AMs are actively reprogrammed during preinvasive stages. Mutant epithelial cells secrete AREG to activate EGFR signaling in fibroblasts, which produce tenascin C to act on AMs via TLR4, inducing a tumor‐promoting state. Distinct AM subsets undergo metabolic rewiring with enhanced CPT1A‐mediated fatty acid oxidation, driving angiogenesis. Senescent AMs develop a SASP (elevated p16^INK4a^, CXCR1, SA‐β‐Gal) to drive immune evasion.

During the acute phase, AMs are susceptible to environmental stressors. For instance, respiratory exposure to 6PPD‐quinone substantially increased CD11c^+^ CD11b^+^ AM populations with impaired chemotactic function, characterized by reduced CCL2, CXCL1, and CXCL2 expression. These altered subpopulations of AMs ultimately exacerbated the severity of *Klebsiella pneumoniae* pneumonia [241]. Following the resolution of primary infections such as sepsis, TrAMs often undergo profound reprogramming driven by localized secondary signals rather than the initial pathogen, resulting in a prolonged paralyzed state exhibiting poor phagocytosis that can persist for months [67]. Specifically, postsepsis AT2 cells release exosomes enriched with lncRNA Rmrp, which are subsequently internalized by AMs. Rmrp stabilizes ZFP36, leading to the targeted decay of Pfkfb3 mRNA. The resulting depletion of PFKFB3, a critical rate‐limiting enzyme for glycolysis, severely impairs aerobic glycolysis in AMs [11]. This metabolic reprogramming forces AMs into a lasting state of immune tolerance and impaired phagocytosis, ultimately rendering the lung highly vulnerable to secondary bacterial pneumonia.

In severe viral pneumonia, AMs exhibit phase‐dependent plasticity. As observed in the acute phase of SARS‐CoV‐2 infection, hampered by peroxisome deficiency and lipid metabolic dysfunction, AMs fail to produce sufficient antiviral IFNs. Instead, they overproduce proinflammatory cytokines such as IL‐1β via ROS‐mediated inflammasome activation, exacerbating ALI and impeding early epithelial defense [242, 243]. During the resolution phase, AMs adopt a tissue‐regenerative phenotype. Following IAV‐induced depletion of TrAMs, recruited BMDMs undergo a spatiotemporal transition to replenish the alveolar pool. These regenerative AMs upregulate and shed Plet1 into the alveolar space. As a crucial paracrine mediator, soluble Plet1 activates the Raf–MEK–ERK signaling pathway and suppresses Src kinase in AECs. This cascade drives epithelial progenitor proliferation and upregulates essential tight junction proteins (ZO‐1, Claudin‐1, and occludin), thereby actively promoting the resealing of the epithelial barrier and the restoration of pulmonary homeostasis [153]. Strikingly, after complete recovery, studies on post‐SARS‐CoV‐2 models reveal that these replenished AMs undergo profound epigenetic reprogramming to establish beneficial innate immune memory. By shifting chromatin accessibility toward IFN‐related pathways and restricting proinflammatory NF‐κB, Memory AMs are prepared for future pathogens and stimuli, initiating a balanced response to clear the virus while limiting excessive inflammation [244]. This temporal evolution underscores that AMs shift from driving acute inflammation to promoting tissue repair, and eventually to providing long‐term protection, thereby influencing disease progression, resolution, and outcomes.

### 5.2 Asthma

Asthma is a heterogeneous disease characterized by airway mucosal abnormalities, intermittent wheezing, and chronic inflammation [270, 271]. Interactions between immune system development and microbial dysbiosis drive asthma pathogenesis and heterogeneity [272], with AMs acting as central regulators of airway inflammation and remodeling (Figure 3B) [76].

Intrinsically, AMs possess a baseline capacity to suppress allergic responses. For instance, the targeted depletion of AMs in adult mice markedly increases the severity of experimental allergic airway disease [273, 274]. Following respiratory viral infection, ILC2s exhibit a reduced ability to respond to dust mites and produce Type 2 cytokines and secrete GM‐CSF to drive the differentiation of MoAMs that confer long‐term protection against asthma [275]. The regulatory function of AMs is governed by sophisticated molecular checkpoints. Under homeostatic conditions, TRIM33 binds to the *Ccl2* promoter in AMs to suppress its transcription. In HDM‐induced asthma models, the specific deletion of TRIM33 in AMs results in their hyperactivation, characterized by upregulated MHC II and CD11b expression, and a subsequent surge in CCL2 production. This elevation promotes the influx of inflammatory CD11b^+^ dendritic cells into the lung and draining lymph nodes, which sequentially amplifies Th2 cell priming, eosinophilic inflammation, IgE production, and the release of Th2 cytokines (IL‐4, IL‐5, IL‐13). Adoptive transfer of wild‐type AMs effectively reverses this aggravated phenotype, cementing the role of intrinsic TRIM33 in restricting AM overactivation and CCL2‐mediated immune cell influx [245].

However, continuous stimulation within the asthmatic microenvironment can transform AMs into potent drivers of pathophysiology. IgE‐activated AMs amplify inflammation by releasing cytokines (IL‐1, TNF‐α, IL‐6, GM‐CSF) that prolong eosinophil survival and activate endothelial cells [246]. Additionally, AM‐derived histamine‐releasing factors sustain mast cell and basophil activation, driving the late‐phase asthmatic response [247]. Furthermore, AMs are a major source of cysteinyl leukotrienes (LTC4, LTD4, LTE4), lipid mediators that critically exacerbate airway pathology by inducing mucus hypersecretion, increasing microvascular permeability, impairing ciliary clearance, and disrupting neuronal function [248, 249].

The asthmatic microenvironment can reprogram AMs, particularly impairing their antiviral capacity and predisposing patients to severe virus‐induced asthma exacerbations. In allergic asthma, TSLP‐driven upregulation of Cullin5 (CUL5) in TrAMs triggers the proteasomal degradation of O‐GlcNAc transferase (OGT). The resulting loss of mitochondrial antiviral‐signaling protein (MAVS) O‐GlcNAcylation blocks retinoic acid‐inducible gene I (RIG‐I) antiviral signaling and IFN‐β production, allowing uncontrolled viral replication and driving neutrophilic asthma exacerbations [250]. Collectively, AMs exhibit high plasticity in asthma, functioning both as essential disease restrictors via TRIM33 and as drivers of exacerbations via TSLP‐mediated reprogramming.

### 5.3 Chronic Obstructive Pulmonary Disease

In COPD, AMs drive persistent airway inflammation because of their failure to clear pathogens and apoptotic cells, a defect that fundamentally stems from the loss of intrinsic metabolic plasticity [276]. Specifically, AMs shift into a state of metabolic exhaustion, characterized by a severe impairment of OXPHOS (Figure 3C). This bioenergetic collapse is primarily driven by the downregulation of the NRF2‐regulated malic enzyme 1 (ME1), which disrupts intracellular redox balance and depletes TCA cycle intermediates. Consequently, their over‐reliance on inefficient glycolysis deprives AMs of the essential energy and antioxidant capacity required for effective pathogen clearance and inflammation resolution [14].

The genesis of this metabolic exhaustion is deeply intertwined with external pathogenic stimuli, predominantly CS and systemic lipid disturbances. Initially, CS exposure disrupts AM homeostasis by shifting their metabolic profile away from OXPHOS toward highly active, yet unsustainable, glycolysis [277]. This pathogenic metabolic reprogramming is initiated by the CS‐induced suppression of the SIRT1/MKP‐1 axis, which removes the inhibitory constraints on inflammatory cascades and leads to the hyperactivation of the MAPK and NF‐κB pathways [251].

Furthermore, under the conditions of systemic dyslipidemia, this metabolic derangement could be exacerbated by the aberrant accumulation of circulating cholesterol within AMs. The cholesterol overload severely blunts mitochondrial respiration and triggers massive ROS leakage, which activates the PPIA/NF‐κB cascade to lock AMs into a proinflammatory M1 phenotype secreting excessive IL‐1β, TNF‐α, and IL‐6. Consequently, AM phagocytosis is structurally and energetically paralyzed by NF‐κB‐mediated CD206 downregulation, which impairs target recognition, and by mitochondrial ATP depletion, which prevents target engulfment [252].

These dysfunctional AMs may evade physiological clearance mechanisms to amplify the inflammatory crisis. Driven by the dysregulation of PPAR‐γ and compromised ISM1/csGRP78‐mediated apoptosis, these cells bypass normal cellular senescence (evidenced by p21 downregulation) and adopt an apoptosis‐resistant, hyperactive state [253, 254]. Ultimately, this metabolically exhausted yet hyper‐secretory AM subpopulation perpetuates persistent airway inflammation and unleashes an unabated proteolytic assault (predominantly via MMP‐12), driving the irreversible ECM degradation and alveolar destruction characteristic of progressive emphysema [254].

In summary, while the pathogenesis of COPD involves complex cellular crosstalk within the alveolar niche or in the airway and requires further investigation, it is now evident that COPD is heavily driven by AM dysfunction [278]. Metabolic and epigenetic AM reprogramming possesses great potential for future COPD treatments.

### 5.4 Cystic Fibrosis

Cystic fibrosis (CF) is a multisystem genetic disorder inherited in an autosomal recessive manner, caused by mutations in the cystic fibrosis transmembrane conductance regulator (CFTR) gene, which is located on the long arm of chromosome 7 [279]. Defective CFTR function particularly affects respiratory homeostasis. Dysfunction of CFTR in airway epithelial cells causes dehydration of airway surface liquid and thickening of mucus secretions, which impairs effective mucociliary clearance, leading to lifelong recurrent infections with harmful bacteria (Figure 3D) [280].

Beyond the respiratory epithelium, CFTR is also expressed in mouse and human AMs [281]. Driven by the continuous inflammatory milieu of diseased airways, the recruitment of classical monocytes to airway tissues is markedly amplified [282], and macrophages in the airways of patients with CF are predominantly of monocyte origin [283]. CFTR‐mutated AMs exhibit profound intrinsic immunological defects [284, 285]. For example, CFTR is vital to pH regulation in the phagosomes of mouse AMs [281], and CFTR‐deficient macrophages fail to properly execute phagosome–lysosome fusion and subsequent lysosomal acidification, resulting in a bacterial clearance defect [255, 256]. This intracellular survival of *S. aureus* within AMs primes the airway niche for the colonization of opportunistic pathogens such as *P. aeruginosa* [286]. Consistent with the above findings, exposure of AMs to the serum of CF patients has been shown to negatively affect their ability to phagocytose *P. aeruginosa* [287]. These defects in phagocytosis and lysosomal acidification directly impair pathogen clearance, perpetuating chronic bacterial infections in CF patients.

Furthermore, CFTR dysfunction drives a hyperinflammatory phenotype in AMs, leading to increased proinflammatory cytokine secretion [288]. In response to TGF‐β, AMs in CFTR‐deficient mice release more IL‐6 than wild‐type mice [257]. Similarly, CFTR silencing in human AMs triggers elevated IL‐8 secretion [258]. AMs exacerbate cytokine production via the upregulation of X‐BOX binding protein‐1 (XBP‐1) and induce intense inflammation in the airways of CF patients [259]. It has also been shown that CFTR mutations impair Type I IFN signaling in AMs [282]. Trapped in chronic obstructive CF airways, AMs are continuously exposed to inflammatory stimuli, resulting in unresolved immune activation of the inflammatory response and causing lung injury [259]. Collectively, CFTR dysfunction in AMs disrupts phagocytosis, exacerbates proinflammatory cytokine secretion, and perpetuates chronic inflammation, driving lung damage and pathogen persistence in CF.

### 5.5 Idiopathic Pulmonary Fibrosis

IPF is an ILD caused by excessive collagen accumulation and is characterized by progressive sclerosis of the lungs and a decline in gas exchange capabilities [185]. Recently, there has been mounting evidence highlighting MoAMs as the primary drivers in the development of IPF (Figure 3E). The infiltration of MoAMs could be attributed to the increasing need for repair in the lungs of IPF patients [261]. Fibroblasts and AMs are vital to IPF progression [186, 187]. In advanced stages of IPF, activated macrophages and myofibroblasts mutually stimulate each other, perpetuating a cycle that exacerbates pulmonary fibrosis [62]. Mechanistically, the profibrotic function of MoAMs is governed by a dual regulatory system involving both external reciprocal signaling and internal transcriptional networks.

Externally, the CXCL12/CXCR4 axis is the core pathway in their interactions. Fibroblast‐derived CXCL12 bound to CXCR4 on MoAMs and subsequently triggered the activation of the MEK/ERK pathway, which has been proved to induce chemotaxis of MoAMs and influence reparative properties of macrophages [260]. In vitro, the blockade of the MEK/ERK pathway in MoAMs resulted in the marked reduction of collagen and α‐SMA [289]. Preclinical research has also confirmed that the inhibitors of the ERK signaling pathways (hydroxychloroquine) could alleviate pulmonary fibrosis [290]. This crosstalk between AMs and fibroblasts highlights their central role in driving fibrotic progression in IPF.

Internally, MoAMs are characterized by robust expression of genes associated with tissue repair and ECM remodeling, thereby amplifying the output of profibrotic signaling. A defining characteristic of these cells is the marked upregulation of TREM2, a receptor significantly elevated in IPF patients. Sphingomyelin in the alveoli could activate TREM2, conferring a critical survival advantage by protecting MoAMs from apoptosis. Consequently, these persistent macrophages adopt a robust profibrotic phenotype, driving fibrosis by secreting profibrotic mediators like SPP1 and MMP12 and inhibiting the regeneration of AT2 cells [64]. In parallel, the PPARG/SPP1/CD44 signaling pathway is crucial for understanding the development of fibrosis in IPF [261]. It is observed that IPF patients demonstrated upregulated SPP1 expression in MoAMs compared with those in healthy controls [262]. SPP1, a multifunctional glycoprotein implicated in a variety of cell types, is found to support the recruitment and proliferation of monocytes [291]. MoAMs that showed higher SPP1 expression levels exhibited higher metabolic activities than TRMs in IPF patients, featuring the upregulation of APOE. A higher proportion of MoAMs that showed higher SPP1 expression levels correlated with a lower forced vital capacity in IPF patients [261]. Also, MoAMs in IPF patients downregulated *PPARG*, which was identified as a negative transcriptional regulator of SPP1 that deterred it from interacting with CD44. The loss of PPAR‐γ allowed the subsequent intercellular signaling that promotes fibrosis via the epithelial–mesenchymal transition (EMT) pathway [262]. Thus, it is speculated that SPP1 and PPAR‐γ could act as valuable biomarkers for clinicians to predict survival outcomes and assess the stages of fibrosis.

### 5.6 Lung Cancer

Lung cancer, predominantly non‐small cell lung cancer (NSCLC), is a leading cause of cancer mortality globally [292, 293]. Within the tumor microenvironment (TME), TRMs are the primary source of tumor‐associated macrophages (TAMs) [294]. AM‐derived TAMs exhibit profound plasticity, typically transitioning into a protumorigenic phenotype (Figure 3F). For example, in EGFR‐mutant lung adenocarcinoma, AMs downregulate antigen‐presenting markers (CD40, CD80, and CD86) and hypersecrete tumor‐promoting cytokines (CXCL1, CXCL2, and IL‐1) [263].

AMs do not merely react to established tumors, but are actively reprogrammed during preinvasive stages to construct a permissive oncogenic niche. The establishment of this early niche relies on spatiotemporal crosstalk between mutant epithelial cells, fibroblasts, and AMs. In Kras^G12D^‐mutant models, AT2 cells secrete AREG to activate EGFR signaling in fibroblasts. These reprogrammed fibroblasts produce tenascin C, which acts on AMs via TLR4, inducing a tumor‐promoting state characterized by high Msr1 and low MHC II expression. Depletion of these AMs impairs neutrophil and γδ T cell recruitment, halting early tumor growth [265].

Concurrently, distinct AM subsets undergo metabolic rewiring to facilitate vascularization during atypical adenomatous hyperplasia (AAH), a critical precancerous stage. Specifically, an S100a4^+^ AM subpopulation exhibits enhanced CPT1A‐mediated FAO. This metabolic shift confers a proangiogenic signature (elevated ANXA2, VEGF, and HIF‐1α), driving endothelial tube formation and epithelial proliferation. The presence of analogous CD68^+^CD11c^+^S100A4^+^CPT1A^+^ANXA2^+^ macrophages in human AAH tissues highlights the translational potential of metabolic interventions, such as CPT1A inhibition with etomoxir, at the preinvasive stage [141].

Furthermore, senescent AMs drive immune evasion during early tumorigenesis. In *Kras*‐driven models, a subset of TrAMs develops a senescence‐associated secretory phenotype (SASP), marked by elevated p16^INK4a^, CXCR1, and SA‐β‐Gal activity. These cells release factors that suppress the infiltration and expansion of cytotoxic CD8^+^ T cells, thereby orchestrating an immunosuppressive TME. Analogous p16^+^ CXCR1^High^ macrophages are also prevalent in early‐stage human NSCLC [264]. In summary, TrAMs are active architects of the nascent TME. Through mediating fibrotic crosstalk, driving metabolic‐dependent angiogenesis, and orchestrating senescence‐induced immune evasion, AM reprogramming is deeply intertwined with early lung carcinogenesis.

## 6 Novel Therapeutic Approaches Targeting AMs

AMs are key effector cells in first‐line environmental toxin and pathogen defense and lung homeostasis maintenance [295], and modulation of the AMs phenotype and function is a potential tool for drug development and therapy optimization related to lung diseases. To achieve optimal bioavailability, several selective systems that target AMs have been developed. For example, inhaled targeted drug delivery that relies on AM‐specific receptors, such as CD206, reduces first‐pass metabolism in the liver, attenuates side effects, improves patient compliance, and is a promising drug delivery modality for treating lung diseases associated with AMs [296, 297]. In addition, research on ECM components, AM metabolism, AM–pathogen interactions, and immuno‐infectious therapeutic targets is increasing to combat pathogen resistance and improve therapeutic efficiency (Table 2).

**TABLE 2:** Novel therapeutic approaches targeting AMs.

Therapeutic strategy	Active pharmaceutical agent	Target disease	Mechanism	Effects	Current clinical translation status	References
Driving and reprogramming the polarization	R428	Pulmonary fibrosis	Inhibits Gas6/AXL signaling axis → blocks downstream AKT/STAT cascades → blunts TGF‐β1 secretion and prevents myofibroblast activation	Inhibits profibrotic polarization of AMs; ameliorates bleomycin‐induced pulmonary fibrosis	Preclinical	[298]
	TD139	IPF	Competitively blocks Gal‐3 → inhibits TGF‐β/SMAD signaling and integrin activation → reduces downstream pathogenic biomarkers (YKL‐40, CCL18)	Inhibits profibrotic polarization of AMs; alleviates IPF	Phase 1/2a trial	[299]
	hUCMSC‐EVs (miR‐486‐5p)	Pulmonary fibrosis (early inflammatory phase)	Via EV‐mediated miRNA delivery → suppresses NF‐κB signaling → resolves early inflammatory phases	Promotes the shift to a proresolving CD206^+^ AM phenotype; attenuates early‐stage pulmonary fibrosis	Phase 1 trial	[300]
	MExos + miR‐23b	ALI	Binds CD206 receptors → delivers miR‐23b to target *Lpar1* mRNA → suppresses NF‐κB activation → reduces proinflammatory cytokines	Halts pathogenic proinflammatory AM polarization; alleviates sepsis‐induced ALI	Preclinical	[301]
	rhGM‐CSF	ARDS	Activates GM‐CSFR → blunts systemic inflammation	Reprograms AMs toward restorative phenotype; improves pulmonary oxygenation	Phase 2 trial	[302]
Metabolic intervention in AMs	AdMSC‐Exos	ALI	Modulates mitochondrial metabolic program → restores AM metabolic function → suppresses inflammation and enhances bacterial clearance	Rewires mitochondrial program in AMs; reduces lung injury; increases survival rates.	Preclinical	[303]
	4‐PBA	Respiratory viral infection	Enhances peroxisome biogenesis → improves lipid metabolism and reduces mitochondrial ROS → mitigates inflammatory microenvironment	Reduces oxidative stress in AMs; mitigates inflammation/fibrosis and promotes alveolar regeneration	Preclinical	[243]
Intervention of oxidative stress	RES	ASFV‐infected pulmonary injury	Activates NRF2 signaling pathway → upregulates antioxidative genes and GSH levels → scavenges excessive ROS	Activates antioxidant response of AMs; reduces cellular damage and inhibits viral replication	Preclinical	[304, 305]
	Sulforaphane	COPD	Targets NRF2 → upregulates MARCO and NRF2‐regulated antioxidant genes	Improves bacterial phagocytosis and ex vivo clearance of AMs	Preclinical	[306]
	Compound 7	COPD	Selectively inhibits KEAP1–Nrf2 interaction → activates Nrf2 → upregulates antioxidant genes (HO‐1, GCLC, NQO1)	Reverses defective AM phagocytosis; did not induce cytotoxicity	Preclinical	[307]
Modulation of the autophagy–apoptosis balance and other cell death pathways	ZKMG	IPF	Modulates TLR4/MyD88/NF‐κB and related pathways → balances AMs’ apoptosis and autophagy → exerts anti‐inflammatory effects	Balances autophagy/apoptosis in AMs; effectively delays the progression of lung fibrosis	Preclinical	[308]
	BML‐111	Inflammatory lung diseases	Activates LXA4 receptor → inhibits MAPK1/MAPK8 activation → induces autophagy and inhibits apoptosis	Induces autophagy and inhibits apoptosis in AMs; attenuates lung inflammation and tissue damage	Preclinical	[97]
	BSP	ARDS	Modulates PAD4 function → suppresses NETs formation and NLRP3 inflammasome activation → alleviates AMs pyroptosis	Inhibits pyroptosis in AMs; alleviates ARDS	Preclinical	[309]
	Andrographolide	ALI	Binds RAGE receptor → downregulates PI3K/AKT/mTOR signaling → restores autophagic flux	Inhibits NLRP3/Caspase‐1/GSDMD‐mediated pyroptosis; reduces proinflammatory cytokines (IL‐1β, IL‐18); ameliorates lung injury	Preclinical	[211]
Reversing the aging trend	SRT 1720	Pulmonary fibrosis	Inhibits the release of SASP factors → prevents activation of lung myofibroblasts	Suppresses AM senescence; hinders the progression of fibrosis	Preclinical	[310]
	Hyaluronidase	Influenza A infection	Removes accumulated extracellular hyaluronic acid → restores proliferation‐promoting effects of GM‐CSF on AMs	Promotes AM proliferation; accelerates alveolar repair after viral infection	Preclinical	[311]
Regulating trained immunity	BCG vaccination	*Mtb* infection	Induces epigenetic/metabolic reprogramming via CD8^+^ T cell‐derived IFN‐γ → establishes memory AMs	Induces trained immunity in AMs; enhances rapid nonspecific resistance to secondary infections	Preclinical	[66]
	β‐Glucan	*L. pneumophila* infection	Induces mild pulmonary inflammation → drives development of ApoE‐dependent monocyte‐derived AMs	Promotes AM expansion; limits bacterial burden upon secondary pathogen challenge	Preclinical	[52]
Other promising AM‐targeted therapies	M‐IL‐4 (engineered macrophages secreting IL‐4 via pulmonary macrophage transplantation)	ALI/ARDS	Sustained secretion of IL‐4 → activates STAT6 signaling pathway → drives M2 polarization of macrophages (upregulates *Arg‐1*, *Ym‐1*, *Fizz‐1*); suppresses proinflammatory cytokines (TNF‐α, IL‐6, IL‐1β)	Reduces AM necrosis; reduces lung inflammation and improves survival rate	Preclinical	[312]
	BMS202	Lung ischemia–reperfusion injury	Blocks PD‐1/PD‐L1 interaction → prevents Src homology 2 domain‐containing protein tyrosine phosphatase (SHP)1/2 recruitment to PD‐1 → relieves SHP1/2‐mediated inhibition of PI3K/AKT signaling	Suppresses AM polarization toward the proinflammatory phenotype (↓CD11c, ↓CD16, ↓CD86); decreases TNF‐α, IL‐1β, IL‐6; attenuates lung edema and damage	Preclinical	[313]

### 6.1 Driving and Reprogramming the Polarization

Targeted reprogramming of AMs represents a highly attractive therapeutic strategy for both acute and chronic lung diseases, and the optimal phenotypic trajectory is strictly context dependent. For instance, while shifting AMs toward a restorative phenotype facilitates the resolution biology in ARDS, this continuous repair executed by M2‐like AMs becomes inherently pathogenic in pulmonary fibrosis [5]. Thus, successful interventions must focus on disease‐specific, spatiotemporal modulation to restore true alveolar immune homeostasis (Figure 4A).

**FIGURE 4:**
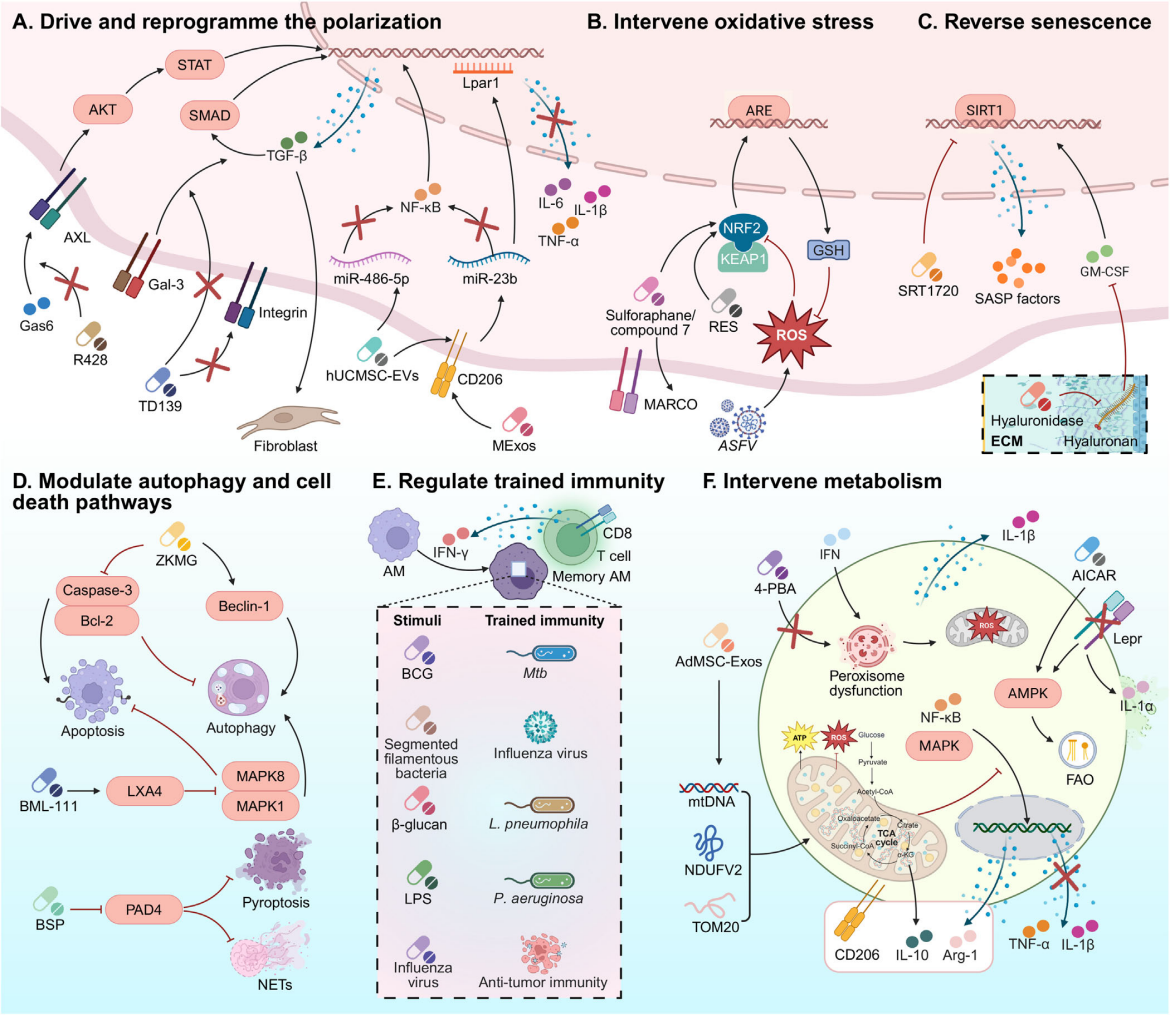
Schematic illustration of novel therapeutic approaches to target AMs. (A) Drive and reprogramme the polarization: Small molecule R428 inhibits AXL to block AKT/STAT cascades, suppressing profibrotic M2 gene transcription. Inhaled TD139 blocks Gal‐3‐mediated TGF‐β/SMAD signaling and integrin activation. Nebulized hUCMSC‐EVs deliver miRNAs (e.g., miR‐486‐5p) to suppress NF‐κB, while MExos deliver miR‐23b to target Lpar1 mRNA and inhibit NF‐κB activation, both promoting proresolving phenotypes. (B) Intervene oxidative stress: NRF2 activators (sulforaphane, compound 7, RES) disrupt KEAP1–NRF2 binding. NRF2 then translocates to the nucleus, binds ARE to regulate antioxidative genes, and increases GSH to scavenge ROS. NRF2 also upregulates MARCO to improve phagocytosis. (C) Reverse senescence: SRT 1720 activates SIRT1, reducing SASP factor release. Hyaluronidase removes accumulated hyaluronan in the ECM, restoring AM proliferation and function. (D) Modulate autophagy and cell death pathways in AMs: ZKMG balances apoptosis and autophagy by adjusting Caspase‐3, Bcl‐2, and Beclin‐1. BML‐111 activates the LXA4 receptor ALX/FPR2 to inhibit MAPK8/MAPK1, promoting autophagy and inhibiting apoptosis. BSP inhibits PAD4 to suppress NETs‐induced pyroptosis. (E) Regulate trained immunity: IFN‐γ from CD8^+^ T cells primes TrAMs into Memory AMs. Various stimuli (BCG, SFB, β‐glucan, LPS, influenza virus) induce trained immunity to enhance resistance against specific pathogens or tumors, or suppress allergic responses. (F) Intervene metabolism: 4‐PBA restores peroxisome function, reversing mitochondrial dysfunction and ROS‐mediated IL‐1β hypersecretion. AdMSC‐Exos transfer mitochondril components or mtDNA/NDUFV2 to restore metabolic fitness. AICAR reactivates AMPK via the Lepr axis to reverse lipid overload and prevent MLKL‐mediated necroptosis and IL‐1α release.

In preclinical and clinical settings, targeting specific signaling axes to halt profibrotic polarization of AMs has shown robust efficacy. For example, the Gas6/AXL signaling axis drives profibrotic M2 polarization in Mo‐AMs, and preclinical models have revealed that inhibiting AXL with small molecules (e.g., R428) blocks downstream AKT/STAT cascades, which suppresses M2‐associated gene transcription (*Arg1*, *CD206*) and blunts TGF‐β1 secretion. By depriving local lung fibroblasts of these essential paracrine signals, AXL inhibition significantly attenuates profibrotic MoAM polarization, and prevents myofibroblast activation and ECM deposition, thereby ameliorating bleomycin‐induced pulmonary fibrosis [298]. Yet, the transition of such systemic small‐molecule inhibitors to clinical application requires rigorous lead optimization to achieve therapeutic efficacy without causing systemic off‐target effects [298]. To circumvent systemic toxicity, localized respiratory delivery provides a highly feasible translational route.

Validating this localized approach, a Phase 1/2a trial of TD139, an inhaled galectin‐3 (Gal‐3) inhibitor, demonstrated robust target engagement in IPF. Administered via a dry powder inhaler, TD139 competitively blocks Gal‐3‐mediated TGF‐β/SMAD signaling and integrin activation on AMs by achieving lung concentrations 500‐fold above systemic levels. This disrupts profibrotic alternative polarization, safely reducing downstream pathogenic biomarkers like YKL‐40 and CCL18 [299]. Similarly, a Phase I trial demonstrated that nebulized human umbilical cord MSC‐derived extracellular vesicles (hUCMSC‐EVs) safely target the alveolar niche to ameliorate pulmonary fibrosis. To resolve the early inflammatory phases that often precede irreversible fibrosis, AMs were mechanistically reprogrammed toward a proresolving M2 (CD206^+^) phenotype via the delivery of immunomodulatory miRNAs that suppress NF‐κB signaling [300].

Despite these promising early‐phase clinical successes, the therapeutic reprogramming of AMs is highly disease‐context dependent, which complicates broad clinical application. While inhibiting M2 polarization is beneficial in progressive fibrosis, promoting a restorative M2‐like phenotype is often the objective in acute inflammatory settings like ARDS. Mannose‐modified exosomes (MExos) enable targeted delivery of miR‐23b to AMs by binding to CD206 receptors. Once internalized, miR‐23b directly targets Lpar1 mRNA, suppressing NF‐κB activation and reducing proinflammatory cytokines (IL‐1β, IL‐6, TNF‐α). This inhibits M1 macrophage polarization and alleviates sepsis‐induced ALI, offering a promising strategy for inflammatory lung diseases [301]. A Phase II trial of intravenous rhGM‐CSF in sepsis‐induced ARDS reprogrammed AMs toward an M2 phenotype, significantly improving pulmonary oxygenation and blunting systemic inflammation [302].

### 6.2 Metabolic Intervention

Modern immunometabolism has established that macrophage activation states are tightly coupled to dynamic cellular metabolic reprogramming, specifically the transitions between glycolysis, OXPHOS, and lipid metabolism [314]. Consequently, intervening pathways that govern AM metabolic flux are indispensable for developing targeted therapies aimed at resolving lung inflammation by recalibrating AM functionality (Figure 4F) [315, 316].

Dysfunctional AMs with aberrant mitochondrial activity are considered to underlie severe lung inflammation [317]. Recent studies demonstrate that exosomes derived from adipose‐derived mesenchymal stem cells (AdMSC‐Exos) can effectively intervene in lung inflammation and injury by modulating the mitochondrial metabolic programs of macrophages. In endotoxin‐treated mice, AdMSC‐Exos exhibited immunomodulatory and lung‐protective properties [303]. These findings highlight the potential of stem cell‐derived exosomes to restore AM metabolic fitness and suppress hyperinflammation in acute lung diseases.

Peroxisomes are vital but often overlooked metabolic organelles. Recent evidence highlights that during severe respiratory viral infections, IFN‐driven loss of peroxisomes in AMs causes profound mitochondrial dysfunction and ROS‐mediated IL‐1β hypersecretion. Pharmacologically restoring AM peroxisomal function with 4‐PBA effectively reverses the metabolic collapse, resolving hyperinflammation, clearing dysplastic KRT8^+^ cells, and promoting functional alveolar repair to prevent postviral sequelae [243]. Normally, Lepr signaling maintains calcium‐dependent AMPK activation to prevent lipid overload. In Lepr‐deficient AMs, lipid overload induces MLKL‐mediated necroptosis, triggering massive IL‐1α release that exacerbates lung inflammation via severe neutrophil recruitment. Crucially, pharmacological reactivation of AMPK using small‐molecule activators like AICAR can effectively reverse lipid accumulation, prevent AM necroptosis, and resolve subsequent hyperinflammation [136].

### 6.3 Intervention of Oxidative Stress

A growing number of studies have shown that oxidative stress is related to lung diseases, and advanced therapies should focus more on alleviating oxidative stress in AMs. Oxidative stress can cause cellular damage and exacerbate disease severity [318]. NRF2 is a transcription factor that induces the expression of endogenous antioxidative enzymes under oxidative stress conditions [319]. However, conformational changes in Kelch‐like ECH‐associated protein 1 (KEAP1) triggered by the improved intracellular ROS level would disrupt its binding to NRF2 and cause proteasomal degradation [320]. NRF2 has become a popular target for maintaining cellular redox balance (Figure 4B).

As an NRF2 activator, RES reversed the inhibition state of the NRF2 signaling pathway in ASFV‐infected AMs [304]. Thus, NRF2 could bind to the antioxidant response element (ARE) in the nucleus to regulate the expression of antioxidative genes and then improve the GSH level in ASFV‐infected AMs [305]. GSH effectively scavenged excessive ROS induced by ASFV infection, reducing cellular damage and inhibiting viral replication.

COPD patients exhibit defective bacterial phagocytosis in AM [321], and it is generally believed that oxidative stress plays an important role [322]. The transcription factor NRF2 can reverse defective regulatory phagocytosis in COPD, providing a potential therapeutic approach for COPD. In AMs [323], NRF2 and its transcriptional activity decrease with increasing severity of COPD. Targeting NRF2 with pharmacological agents such as sulforaphane to upregulate scavenger receptor (MARCO) and other NRF2‐regulated antioxidant genes was an effective method to improve bacterial phagocytosis and ex vivo clearance of AMs in COPD patients [306]. Notably, the conditioning effect of *S. pneumoniae* could activate the stress transcriptional response of normal AM cells and enhance phagocytosis in response to antioxidant responses, but there is no corresponding effect on the AMs of COPD patients. Fortunately, the selective NRF2 agonist compound 7 reversed defective AMs phagocytosis in COPD patients [307]. Thus, NRF2 agonists, especially those with the Kelch structural domain [324] and the BTB structural domain [325] of KEAP1, which bind and block NRF2 ubiquitination and proteasomal degradation, are highly selective drugs that modulate oxidative stress in COPD and other diseases. These agents represent promising strategies to restore AM phagocytic function and reduce COPD‐related lung damage via antioxidant pathways.

### 6.4 Modulation of the Autophagy–Apoptosis Balance and Other Cell Death Pathways

The promotional or inhibitory effects of autophagy on AM apoptosis depend on the disease pattern and microenvironmental stimuli [326, 327]. As autophagy is a major adaptive response that protects cells and organisms from injury, therapeutically recalibrating this delicate autophagy–apoptosis balance has emerged as a highly promising strategy to curtail tissue damage and slow the progression of pulmonary diseases (Figure 4D) [328].

In chronic lung diseases like IPF, deficient autophagy and excessive apoptosis in AMs promote fibroblast proliferation and differentiation, ECM deposition, and ACE apoptosis, resulting in the acceleration of fibrosis [329]. Restoring the balance between autophagy and apoptosis in AMs thus may become a crucial entry point for the treatment of IPF. As one of the representative Uyghur proprietary medicines, Zukamu granule can balance the apoptosis and autophagy of AMs through multiple cascades, including the IL‐17, TNF, Th17, and TLR4/MyD88/NF‐κB signaling pathways, thereby exerting prominent anti‐inflammatory effects and effectively delaying the process of lung fibrosis [308].

Similarly, in acute inflammatory settings, endogenous lipid mediators, such as LXs, play a pivotal role in resolving pulmonary inflammation via the G‐protein‐coupled LXA4 receptor [95]. Within the alveolar niche, autocrine LX secretion by AMs enhances the phagocytic clearance of apoptotic neutrophils [96]. For instance, the LXA4 agonist BML‐111 robustly attenuates ALI by simultaneously inducing cytoprotective autophagy and inhibiting AM apoptosis. Mechanistically, BML‐111 orchestrates this cellular balance by specifically suppressing the hyperactivation of MAPK1 and MAPK8 in a strictly mTOR‐independent manner [97].

Further illustrating the complexity of AM cell fate modulation, other forms of programmed cell death are also worth being discussed. *Bletilla striata* polysaccharides (BSP) were reported to alleviate ARDS by influencing NETs and pyroptosis in AMs [309]. In vitro, NETs could induce pyroptosis in AMs synergistically with LPS. Their presence resulted in significantly higher expression levels of NLRP3, Caspase‐1, GSDMD, IL‐6, and TNF‐α which played a role in the pyroptosis and inflammasome pathway. Notably, the attenuating effect of BSP was counteracted when PAD4 ablation was introduced by GSK484, indicating the participation of PAD4 in the effects. PAD4 functioned as a proinflammatory mediator by mediating the formation of NETs and activating the NLRP3 inflammasome. Interestingly, the coadministration of BSP and GSK484 did not achieve a significant improvement, while each treatment independently suppressed NET formation and pyroptosis. One possible explanation for this paradox is that GSK484 may potentially upregulate alternative inflammatory pathways, such as PAD2‐dependent NET formation or ROS‐mediated inflammasome activation. PANoptosis, a recently defined programmed cell death integrating apoptosis, pyroptosis, and necroptosis, further drives AM fate determination during severe inflammation. For instance, upregulating TIPE2 has been proved to alleviate sepsis‐induced lung injury by actively suppressing ZBP1/TRIF‐dependent PANoptosis, thereby targeting TIPE2 presents a highly promising strategy to rescue lethal lung damage [330]. These findings highlight the complexity of targeting AM death pathways in therapeutic settings, and emphasize the importance of precisely orchestrating the broader autophagy–apoptosis and cell survival networks is imperative to achieve expected benefits in fibrosis and inflammation treatment.

### 6.5 Reversing the Aging Trend

As detailed in previous sections, AMs undergo distinct state transitions with natural aging or severe infections, leading to the accumulation of senescent AMs. Cellular senescence is characterized by the secretion of SASP factors, such as cytokines, growth factors, matrix‐degrading enzymes, and developmentally relevant molecules, which are implicated in the development and progression of various fibrotic conditions, embryonic patterns, organogenesis, tumor suppression, and tissue repair processes [331]. The persistent accumulation of senescent cells in IPF may exacerbate fibrosis, and macrophages exhibit characteristics similar to those of senescent cells, particularly their secretory proinflammatory phenotype and cell cycle arrest, making them potential therapeutic targets for IPF (Figure 4C) [332]. For example, by activating Sirt1, SRT 1720 could affect the release of SASP factors from AMs and inhibit the activation of lung myofibroblasts, further hindering the progression of pulmonary fibrosis [310].

TrAMs recovered from acute respiratory *S. pneumoniae* infection show features of cell senescence and reduced proliferation capacity [50]. Age‐related reductions in AM numbers or diminished function may partly account for the increased susceptibility of elderly individuals to pneumonia. A significantly increased hyaluronic acid content in the ECM of aging individuals inhibits the proliferation‐promoting effects of GM‐CSF on MoAMs [57]. The ability of hyaluronidase, which can remove accumulated hyaluronic acid, to accelerate repair and restore lung function has been demonstrated in a mouse model of influenza A infection [311]. Collectively, reversing the aging trend via interventions at both the cellular and microenvironmental levels could effectively restore their functional capacity and improve outcomes in aging‐related lung diseases.

### 6.6 Regulating Trained Immunity

Trained immunity refers to the biological process by which innate immune cells undergo persistent functional reprogramming through epigenetic modifications and metabolic reprogramming following initial exposure to stimuli, thereby enhancing nonspecific immune responses upon secondary challenges. Although memory‐like functionality following BCG vaccination was first observed in 2003 [333], the conceptual framework of trained immunity was formally established in 2011 [334]. This process involves multidimensional regulatory mechanisms, including functional reshaping of immune cells, epigenetic reprogramming, metabolic reprogramming, and activation of signaling pathways [335, 336, 337]. The initiation of memory AMs requires assistance from effector CD8^+^ T cells, which trigger this process via IFN‐γ secretion [65]. This mechanism reveals a novel bridge between innate and adaptive immunity: adaptive T cells program mucosal macrophages for innate memory, enabling rapid and robust immune responses against secondary infections. Researchers are actively exploring strategies to translate trained immunity of AMs into innovative preventive and therapeutic approaches for respiratory diseases (Figure 4E).

Recent advances have uncovered multiple pathways to enhance AMs’ function via trained immunity. Subcutaneous BCG vaccination independently induces memory AMs targeting *Mtb* infection [66]. Commensal segmented filamentous bacteria reprogram AMs, improving influenza virus neutralization and phagocytic function while maintaining inflammatory tolerance to better protect the lungs [88, 338]. Intranasal β‐glucan exposure induces mild pulmonary inflammation, driving the development of ApoE‐dependent MoAMs (ApoE^+^ CD11b^+^ AMs), which subsequently limit bacterial burden after *Legionella pneumophila* (*L. pneumophila*) infection [52]. LPS exposure expands a MERTKʰ^i^ Marcoʰ^i^ CD163^+^ F4/80ˡ°ʷ TrAM subset with enhanced phagocytic capacity, protecting mice from infections by pathogens such as *P. aeruginosa* [127]. Influenza‐trained AMs can also be further trained to enhance tumor cell cytotoxicity, eliciting sustained and tissue‐specific antitumor immunity [339].

### 6.7 Current Translational Status of AM‐Centric Therapies

Besides the above strategies discussed, emerging therapies targeting AMs include lung macrophage transplantation [312], therapeutic dietary interventions [340], and immune checkpoint blockade [313]. Moreover, synergistic AM‐centric strategies, such as combining macrophage transplantation with gene editing or immune training, could unlock novel therapeutic breakthroughs for intractable pulmonary diseases, particularly severe inflammation and cancer.

Murine models are foundational for respiratory research, and evaluating the cross‐species conservation of AMs is essential for clinical translation. Murine and human AMs share a core phenotypic identity characterized by the conserved expression of surface markers such as CD11c, CD206, CD64, and MARCO, alongside a shared dependence on PPARγ‐driven lipid metabolism to adapt to the surfactant‐rich alveolar niche [48, 59]. Furthermore, recent single‐cell transcriptomics reveal that specific murine AM subpopulations are faithfully mirrored in human airways, including a self‐renewing proliferating subset driven by *Top2a* and *Mki67*, and a proinflammatory subset characterized by the enriched expression of *CD63* and *Fcer1g* [48, 54]. Metabolically distinct subpopulations, such as a Zeb2‐expressing AM cluster characterized by enhanced OXPHOS and higher mitochondrial content, are also highly conserved across both species [54]. Despite these profound functional similarities, notable interspecies differences exist. For instance, *SiglecF* is exclusively used to identify murine AMs, whereas human AMs uniquely rely on CD169, express the C1Q gene which is strictly restricted to IMs in mice, and contain a rare, human‐specific metallothionein‐expressing subpopulation [54, 59]. Nevertheless, the strong transcriptomic and functional alignment of the major AM subsets, particularly regarding their inflammatory and metabolic states, provides robust evidence that targeting these specific AM populations in murine preclinical models holds significant translational value for human pulmonary diseases.

Also, recent studies elucidate novel metabolic mechanisms of AMs, providing new targets for future preclinical research. AM‐targeted therapies must account for their specific metabolic traits. For example, although widely recognized as an anti‐inflammatory metabolite in BMDMs, itaconate promotes NLRP3 inflammasome activation in TrAMs, arising from the low‐glucose alveolar microenvironment that requires AMs to rely on OXPHOS for energy. When itaconate inhibits SDH, AMs adaptively enhance the activity of mitochondrial ETC‐CI to maintain OXPHOS. Consequently, this compensatory ETC‐CI overactivation directly triggers the NLRP3 inflammasome, leading to excessive IL‐1β release and exacerbated ALI. These findings highlight that treatment with ETC‐CI inhibitor holds potential for relieving ALI [209]. Another study revealed that loss of leptin receptor signaling impairs calcium‐dependent AMPK activation, leading to lipid overload. This triggers MLKL‐mediated necroptosis and subsequent IL‐1α release, which exacerbates lung inflammation through extensive neutrophil recruitment. Pharmacological reactivation of AMPK using small‐molecule activators effectively reverses this lipid accumulation and prevents AM necroptosis [136]. The accumulation of these metabolic and organelle stresses frequently shifts AMs toward broad, interconnected cell death networks. Recent studies highlight the role of PANoptosis, an integrated form of cell death encompassing apoptosis, pyroptosis, and necroptosis. For instance, upregulating TIPE2 alleviates sepsis‐induced lung injury by suppressing ZBP1/TRIF‐dependent PANoptosis in AMs [330]. Preclinical therapeutic strategies aiming at this pathway could prioritize the development of TIPE2 mimetics to shut down this inflammatory cascade.

Encouragingly, several AM‐centric therapies for pulmonary diseases have already advanced into clinical trials (Table 3). Early‐phase evaluations of some strategies have yielded positive outcomes, particularly regarding safety and target engagement. For example, the administration of hUCMSC‐EVs demonstrated safety and significantly improved lung function (FVC and MVV) in pulmonary fibrosis patients by delivering miRNAs that suppress early AM‐mediated inflammatory signaling [300]. Similarly, in a Phase 1 trial, the antimicrobial prodrug SPR720 achieved highly targeted intracellular accumulation specifically within AMs, effectively penetrating the cellular niche where pathogenic *mycobacteria* reside [341].

**TABLE 3:** Clinical trials involving AMs in pulmonary diseases.

Mechanism category	NCT/ChiCTR No.	Objective target disease	Active pharmaceutical agent	Effect on AMs	Preliminary findings	Current clinical translation status	References
Intracellular accumulation	NCT05955586	NTM‐PD (due to MAC)	SPR720 (prodrug of SPR719)	Accumulates highly within AMs to inhibit DNA gyrase B of internalized *mycobacteria*	Positive Safety: well‐tolerated (mild TEAEs) Efficacy: excellent AM penetration	Phase 1 trial	[341]
	NCT03910673	Lower respiratory tract bacterial infections (MDR pathogens like CRE)	Intravenous fosfomycin	Penetrates into the ELF and intracellularly into AMs	Positive Safety: well‐tolerated (mostly mild TEAEs, no SAEs) Efficacy: effective AM penetration	Phase 1 trial	[342]
	NCT04710407	Lower respiratory tract bacterial infections	Tebipenem pivoxil hydrobromide	Distributes into lung compartments and penetrates AMs	Positive Safety: well‐tolerated Efficacy: quantifiable penetration into ELF and AMs	Phase 1 trial	[343]
Phenotype reprogramming	ChiCTR2300075466	Pulmonary fibrosis (early inflammatory phase)	hUCMSC‐EVs (miR‐486‐5p)	Delivers miR‐486‐5p to suppress NF‐κB signaling	Positive Safety: well‐tolerated (no SAEs) Efficacy: improved lung function and respiratory scores	Phase 1 trial	[300]
	NCT02257177	IPF	TD139	Inhibits Gal‐3 to block TGF‐β/SMAD signaling and integrin activation	Positive Safety: well‐tolerated; Efficacy: concentration‐dependent reduction of Gal‐3 on AMs; decreased plasma biomarkers	Phase 1/2a trial	[299]
	NCT03832946	IPF	TD139		Negative Safety: more treatment‐related AEs Efficacy: did not meet primary endpoint (52‐week FVC decline); TD139 group showed a greater mean FVC decline (–316.6 mL) compared with placebo (–127.4 mL)	Phase 2b	[344]
	ChiCTR1800014733	ARDS	rhGM‐CSF	Activates GM‐CSFR/JAK2/STAT5 pathway	Negative: Safety: well‐tolerated (no SAEs) Efficacy: improved pulmonary function; did not significantly impact 28‐day mortality or VAP incidence	Phase 2 trial	[302]
Intracellular homeostasis	NCT02649751	CF (chronically infected with *Pseudomonas aeruginosa*)	Roscovitine (Seliciclib)	Inhibits CDKs to decrease intraphagosomal pH and restore bactericidal activity	Negative Safety: well‐tolerated Efficacy: no significant clinical efficacy	Phase 2a trial	[345]
	NCT01335971	COPD	Sulforaphane	Activates Nrf2 to induce antioxidant gene expression (e.g., NQO1, HO1, AKR1C1, AKR1C3)	Negative Safety: well‐tolerated Efficacy: did not stimulate Nrf2 target gene expression in AMs or bronchial epithelial cells; no significant clinical efficacy	Phase 2 trial	[346]

Abbreviations: AKR1C1, aldo‐keto reductase family 1 member C1; AKR1C3, aldo‐keto reductase family 1 member C3; APACHE II, acute physiology and chronic health evaluation II; ATPase, adenosine triphosphatase; CDK, cyclin‐dependent kinase; CRE, carbapenem‐resistant Enterobacteriaceae; ELF, epithelial lining fluid; FEV1, forced expiratory volume in 1 second; FVC, forced vital capacity; HMGB‐1, high mobility group box 1; HO1, heme oxygenase 1; HRCT, high‐resolution computed tomography; LCQ, Leicester cough questionnaire; MAC, *Mycobacterium avium* complex; MDR, multidrug resistant; MVV, maximal voluntary ventilation; NQO1, NAD(P)H:Quinone oxidoreductase 1; NTM‐PD, nontuberculous mycobacterial pulmonary disease; PAI‐1, plasminogen activator inhibitor‐1; PBP, penicillin‐binding protein; PDGF‐BB, platelet‐derived growth factor BB; SGRQ, St. George's respiratory questionnaire; SOFA, sequential organ failure assessment; VAP, ventilator‐associated pneumonia.

However, despite the therapeutic effects proved by preclinical trials, when advancing into the later stage, some strategies did not achieve estimated efficacy. A Phase 1/2a trial of TD139, an inhaled Gal‐3 inhibitor, demonstrated that dry powder inhaler delivery achieves pulmonary concentrations 500‐fold higher than systemic levels. This competitive blockade of TGF‐β signaling and integrin activation successfully disrupts the profibrotic alternative polarization of AMs, correlating with a reduction in pathogenic biomarkers such as YKL‐40 and CCL18 [299]. Yet, the subsequent Phase 2b trial failed to meet its primary efficacy endpoint. Patients receiving TD139 experienced a greater mean decline in FVC (−316.6 mL) compared with the placebo group (−127.4 mL), alongside a higher incidence of treatment‐emergent adverse events (TEAEs). Similar efficacy failures have also been observed with targets like Nrf2 (sulforaphane) and CDK (roscovitine) [345, 346]. The clinical failure of TD139 may stem from underestimating the complexity of AM plasticity and signaling pathways. Gal‐3 not only drives early fibrogenesis, but is also later required for AM‐mediated efferocytosis and tissue repair [347]. Therefore, sustained Gal‐3 inhibition likely disrupts natural lung healing. Furthermore, prolonged single‐target blockade in IPF often activates compensatory profibrotic pathways, ultimately negating long‐term therapeutic benefits. These clinical outcomes provide insights into the future direction of AM‐centric treatment development. First, the plastic nature of AMs necessitates carefully timed therapeutic strategies to align with the acute or resolution stage of pulmonary diseases. Second, future research should focus on exploring synergistic approaches to cope with the complex signaling networks, or alternatively targeting upstream metabolic or epigenetic targets rather than blocking single downstream effector proteins. Finally, advanced targeted delivery systems should be developed to prevent off‐target effects.

## 7 Perspective

In this review, we have comprehensively summarized the biological insights of AMs, revealing them as central regulators of lung balance. The multifaceted functions of AMs are profoundly shaped by their ontogeny, metabolic profiles, signaling networks, microenvironmental cues, and cell–cell interactions. Reduced cellular abundance or functional impairment of these cells strongly correlates with heightened susceptibility to various pulmonary diseases. Consequently, AMs emerge as promising therapeutic targets, necessitating future research to prioritize precise modulation strategies that restore their homeostatic capacity to rebalance inflammation and effectively treat intractable respiratory disorders.

AM identity and function are profoundly influenced by bidirectional crosstalk with surrounding structural and immune cells within the alveolar niche [348]. AMs and AECs establish a mutualistic symbiosis, wherein AECs sustain AM quiescence via specific cytokines (GM‐CSF, IL‐10), while AMs reciprocate postinjury by utilizing paracrine signals (OSM, RvD1) and direct gap junctions (Cx43) to drive epithelial repair [109, 152, 154]. In addition to AT2 epithelial cells, ILC2s and basophils are other sources of GM‐CSF [109]. Beyond structural cells, AMs continuously coordinate with fibroblasts to drive pathological matrix deposition in fibrosis [165]. These findings highlight that AMs do not act merely as isolated immune sensors, but also connect complex multicellular networks that orchestrate alveolar immunity, tissue remodeling, and immune tolerance.

The delicate balance between pulmonary immune defense and tissue damage relies heavily on highly plastic AM subsets that are shaped by ontogeny, metabolism, and spatial niches, and the dysregulation of these specific phenotypes directly drives distinct pathologies. The traditional dichotomy of proinflammatory M1 and anti‐inflammatory M2 phenotypes is increasingly obsolete, failing to capture the profound in vivo metabolic heterogeneity of AMs. Recognizing this multidimensional plasticity beyond the traditional binary models, we propose a conceptual framework that categorizes AMs into five distinct subtypes: inflammatory AMs, repair/regulatory AMs, memory AMs, senescent AMs, and proliferating AMs. Within specific disease contexts, metabolically reprogrammed inflammatory AMs amplify tissue damage and Type 2 inflammation in acute infections like SARS‐CoV‐2 and chronic conditions such as asthma and COPD via excessive IL‐1β release and IL‐33/ILC2 signaling [62, 243]. While repair/regulatory AMs essentially promote resolution, their spatial dysregulation, particularly within recruited MoAMs, generates pathological profibrotic (TREM2^+^) or tumor‐promoting phenotypes that perpetuate matrix deposition in IPF and lung cancer [64, 134]. Concurrently, epigenetic remodeling and aging spawn memory AMs and senescent AMs that dictate divergent trajectories like protective antiviral memory or postinfection paralysis [58, 65, 66, 67], whereas proliferating AMs independently sustain the resident pool via Wnt/β‐catenin self‐renewal [189, 349]. Given this profound heterogeneity, developing standardized analytical frameworks is essential to translate the intricate granularity of single‐cell omics into consistent diagnostic insights.

Recent single‐cell transcriptomics reveal that specific surface markers are highly conserved between murine and human AMs, and murine AM subpopulations are faithfully mirrored in human airways, including proliferating and distinct proinflammatory subsets [48, 54, 59]. While AM‐targeted clinical trials are at an early stage, this robust cross‐species conservation validates preclinical models as reliable predictors for translating AM‐targeted therapies to humans. From a disease‐specific perspective, modulating the diverse pathophysiological states of AMs provides novel therapeutic avenues across various respiratory disorders. Specifically, eliminating senescent AMs or inducing trained immunity via BCG/β‐glucan effectively targets intractable IPF and recurrent infections respectively [66, 127].

Despite these promising prospects, the clinical feasibility and translation of AM‐targeted therapies hinge heavily on overcoming significant drug delivery challenges. The heterogeneous spatial distribution of AMs, with 60% of alveoli completely lacking them, severely hinders the uniform delivery of therapeutics [78]. Furthermore, the complex pathologic pulmonary microenvironment, including the dynamic autophagy–apoptosis balance influenced by local inflammatory stimuli, can severely impede the efficient cellular uptake and intended efficacy of therapeutics [350, 351].

However, optimizing targeted delivery represents only the first step toward clinical translation. Translating targeted therapies into clinical success is further complicated by the unpredictable downstream consequences of manipulating fundamental AM biological states. First, failed synergies between individually effective agents, such as BSP and GSK484 in ARDS, reveal the complexity of inflammatory networks and the risk of inducing alternative ROS‐mediated pyroptosis [309]. Second, the distinct epigenetic rewiring and metabolic thresholds dictating whether trained immunity confers host protection or entrenches maladaptive, chronic inflammatory memory remain incompletely resolved [352]. Finally, although senolytics and lipid modulation ameliorate experimental fibrosis, the pleiotropic nature of SASPs means systemic interventions risk disrupting physiological tissue repair or promoting untargeted oncogenesis in humans [353].

Future AM‐targeted therapeutic development must pivot from broad, systemic immunosuppression toward precision cellular modulation. Advancing single‐cell and spatial omics will decode in vivo AM heterogeneity, mapping the spatial distribution, state transitions, and communication networks of specific AM subtypes across disease niches [354]. By leveraging these omics‐derived mechanistic insights, researchers can design advanced targeted delivery systems that precisely reprogram specific pathogenic AM subsets, thereby restoring pulmonary homeostasis and offering highly personalized, curative strategies for intractable respiratory disorders.

## Author Contributions

S.Y. and R.L. wrote the manuscript, designed the figures, collected the related references, and edited the manuscript; C.Y. provided guidance and revised this manuscript. X.Z. and J.L. conceived, provided guidance, and revised this manuscript. All the authors approved the final manuscript.

## Ethics Statement

The authors have nothing to report.

## Conflicts of Interest

The authors declare no conflicts of interest.

## Data Availability

The authors have nothing to report.
